# A Theory of Rate Coding Control by Intrinsic Plasticity Effects

**DOI:** 10.1371/journal.pcbi.1002349

**Published:** 2012-01-19

**Authors:** J. Naudé, J. T. Paz, H. Berry, B. Delord

**Affiliations:** 1 Institut des Systèmes Intelligents et de Robotique, CNRS – UMR 7222, Université Pierre et Marie Curie (UPMC), Paris, France; 2 Department of Neurology & Neurological Sciences, Stanford University Medical Center, Stanford, California, United States of America; 3 Project-Team BEAGLE, INRIA Rhone-Alpes, LIRIS UMR5205, Université de Lyon, Lyon, France; Indiana University, United States of America

## Abstract

Intrinsic plasticity (IP) is a ubiquitous activity-dependent process regulating neuronal excitability and a cellular correlate of behavioral learning and neuronal homeostasis. Because IP is induced rapidly and maintained long-term, it likely represents a major determinant of adaptive collective neuronal dynamics. However, assessing the exact impact of IP has remained elusive. Indeed, it is extremely difficult disentangling the complex non-linear interaction between IP effects, by which conductance changes alter neuronal activity, and IP rules, whereby activity modifies conductance via signaling pathways. Moreover, the two major IP effects on firing rate, threshold and gain modulation, remain unknown in their very mechanisms. Here, using extensive simulations and sensitivity analysis of Hodgkin-Huxley models, we show that threshold and gain modulation are accounted for by maximal conductance plasticity of conductance that situate in two separate domains of the parameter space corresponding to sub- and supra-threshold conductance (i.e. activating below or above the spike onset threshold potential). Analyzing equivalent integrate-and-fire models, we provide formal expressions of sensitivities relating to conductance parameters, unraveling unprecedented mechanisms governing IP effects. Our results generalize to the IP of other conductance parameters and allow strong inference for calcium-gated conductance, yielding a general picture that accounts for a large repertoire of experimental observations. The expressions we provide can be combined with IP rules in rate or spiking models, offering a general framework to systematically assess the computational consequences of IP of pharmacologically identified conductance with both fine grain description and mathematical tractability. We provide an example of such IP loop model addressing the important issue of the homeostatic regulation of spontaneous discharge. Because we do not formulate any assumptions on modification rules, the present theory is also relevant to other neural processes involving excitability changes, such as neuromodulation, development, aging and neural disorders.

## Author Summary

Over the past decades, experimental and theoretical studies of the cellular basis of learning and memory have mainly focused on synaptic plasticity, the experience-dependent modification of synapses. However, behavioral learning has also been correlated with experience-dependent changes of non-synaptic voltage-dependent ion channels. This intrinsic plasticity changes the neuron's propensity to fire action potentials in response to synaptic inputs. Thus a fundamental problem is to relate changes of the neuron input-output function with voltage-gated conductance modifications. Using a sensitivity analysis in biophysically realistic models, we depict a generic dichotomy between two classes of voltage-dependent ion channels. These two classes modify the threshold and the slope of the neuron input-output relation, allowing neurons to regulate the range of inputs they respond to and the gain of that response, respectively. We further provide analytical descriptions that enlighten the dynamical mechanisms underlying these effects and propose a concise and realistic framework for assessing the computational impact of intrinsic plasticity in neuron network models. Our results account for a large repertoire of empirical observations and may enlighten functional changes that characterize development, aging and several neural diseases, which also involve changes in voltage-dependent ion channels.

## Introduction

Ion channels of neuron membranes undergo long-term experience-dependent modifications of their biochemical and biophysical state induced by on-going neuronal activity, a process called intrinsic plasticity (IP; [1], [2]). Regulating channels' state changes neuron excitability, i.e. its propensity to discharge in response to synaptic inputs. Thus, IP continuously modifies collective neuronal dynamics, taking part to the adaptive and learning abilities of neural networks, as do synaptic and structural plasticity [3], [4]. Indeed, IP has proved a ubiquitous cellular correlate of behavioral learning [5], [6], [7], [8] and of neural network homeostatic regulation [9], [10], [11]. Conversely, pathological forms of IP can lead to persistent impaired excitability and dysfunctional network behavior, as found in several major CNS disorders [12], [13].

IP involves a causal loop between electrical activity and the channels' state. Indeed, activity-induced signaling pathways modify the channels' state, a process commonly termed IP rules [14]. In turn, channels' state sets the activity in response to synaptic inputs, a dependency we name IP effects hereafter. Depending on their sign, IP loops are though providing distinct computational role to neurons. IP loops with negative feedback may underlie homeostatic regulation of neuronal activity under changing conditions [15], [16]. However, the specific role of homeostatic IP (H/IP) remains to be clarified, compared to other homeostatic processes such as synaptic scaling [17]. In particular, beyond its role on spontaneous neuronal dynamics [18], [19], the impact of H/IP in the frequency domain remains obscure. Indeed, H/IP was proposed 1) to maintain a target frequency [16] but this idea appears at odds with rate coding or 2) to set a target frequency range, but a model testing this hypothesis yields unrealistic frequency–intensity (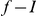) relations [20]. Anti-homeostatic (i.e. positive feedback) IP (AH/IP) loops may support mnemonic processes by maintaining input traces [21], [22]. However, the saturation/silencing dilemma arising from AH/IP remains an open question [23]. Compensatory regulation by H/IP may solve this issue but such interplay between IP forms remains to be assessed [23].

Actually, getting a global picture of the possible computational role of IP loops remains problematic for two reasons. First, IP loops are diverse. IP involves virtually all known ion channel types and many different signaling pathways [1], [22], resulting in a large repertoire of combinations expressed by different neuronal types [1], [24]. As a culminating demonstration, IP was recently demonstrated in vivo to display a striking diversity even within a homogeneous population of pyramidal neurons, with bidirectional excitability changes affecting the threshold or the gain of the 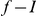, or both [25]. Second, deciphering interactions within the causal loop of IP is methodologically delicate. Indeed, most IP studies monitor a measure (activity) of the entangled influence between neuronal activity and the channels' state, i.e. the interaction between IP rules and IP effects [26], [27], [28], [29], [30]. Interpreting such data is thus problematic as these measures can arise from different combinations of IP rules and IP effects, i.e. the problem is underconstrained. This issue is critical when considering the strong non-linearity of underlying molecular processes [23], [31], which enriches the possible repertoire of dynamical and logical outcomes of the IP loop.

Although the ubiquitous scheme has emerged that electrical activity implicates calcium signaling and kinase/phosphatase pathways to regulate channels' state [22], [32], [33], quantitative data remain scarce to elaborate fine-grained models of IP rules, due to the extreme difficulty of conducting extensive parameterized empirical studies (but see [32], [34]). Hence, most models considering IP in particular neurons are either devoid of IP rule [35], [36] or rely on specific assumptions unlikely met in signaling pathways [37]. By contrast, many models combine specific IP rules and IP effects in order to illustrate a given target computational property [20], [38], [39], [40], [41], [42]. Thus, current models rely on specific assumptions targeted to either account for experimental observation or support theoretical hypotheses.

To avoid such specificities, we propose an alternative modeling strategy devised to provide the generic framework required for a global picture of the IP loop and its functional role. Ideally, this framework should describe IP effects and IP rules with the highest possible generic character. Here, we develop an extensive analysis of generic IP effects on firing rate. This theory is to be combined with generic descriptions of IP rules to enlighten IP loop interactions. In particular, we view the present study as a complement to the aKP model [43], which describes how activity-dependent kinase/phosphatase cycles regulating conductance provide generic properties compatible with those observed for IP rules, such as gradation, rapid (seconds (AH/IP) to hours and days (H/IP)) induction [23] and long-term maintenance [44]. Models combining generic IP effects and IP rules would be ideally suited to overcome the issues encountered in previous study of the IP loop. Indeed, they would 1) address the diversity of IP loops, thanks to their generic character; 2) provide a realistic framework based on molecular properties (e.g. pathways, conductance) 3) unravel the intrinsic entanglement of the IP loop, as both IP rules and IP effects can be independently manipulated in models. Such models would thus represent a powerful mean to assemble a global picture of the possible computational roles offered by IP.

In the following, we perform a sensitivity analysis to systematically quantify how changes in the maximal conductance of a generic voltage-gated conductance affects the threshold and gain of the 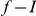. We focus on the 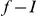 as it is widely used to measure to characterize IP experimentally. Moreover, the 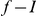 quantifies rate coding, the relevant regime of a vast repertoire of type I excitability neuronal types and an important determinant of asynchronous activity in the awaken state in many CNS structures (e.g. cortices, hippocampus, basal ganglia). Computing the sensitivities across the conductance parameter indicates a generic separation between domains of large threshold versus gain sensitivities, allowing strong inference on the nature of the conductance modified by IP in empirical studies. Besides, we derive analytical descriptions of sensitivities that enlighten the mechanisms governing IP effects in terms of conductance kinetics. Moreover, we show how our results generalize to the IP of kinetic conductance parameters and translate as effective net frequency changes of the neuron discharge. Finally, we consider the example of the homeostatic regulation of spontaneous discharge [45], [46], [47], [48], [49], a neuronal property with strategic computational and metabolic implications [50], [51], to illustrate how our theory of IP effects can enlighten the otherwise unpredictable outcome of IP rules within complex IP loop interactions.

## Results

### A formal analysis of firing rate intrinsic plasticity

We address the issue of how the firing frequency of a neuron, 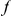, is affected by plastic modifications of a generic voltage-gated membrane ionic conductance that we denote the X conductance. In the classical Hodgkin-Huxley (HH) formalism, conductance properties are specified by biophysical parameters: the maximal conductance 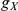 sets its overall quantitative influence while kinetic parameters determine the gating of the conductance (e.g. activation and inactivation voltage-dependences and time constants). In vitro IP experiments point toward the maximal conductance as the most frequently modified parameter by induction protocols [28], [33], [35], [52], [53]. We thus consider plastic modifications of 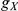 as a representative scheme describing IP (see Parametric exploration in the Methods). However, we show below how IP of other parameters can be derived from that of 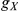.

In this context, the effect of IP on firing rate is captured by the frequency sensitivity, 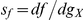, i.e. variations of 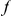 caused by a given 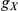 modification. The 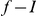 relation, which describes how 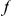 depends on an input current 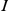 (rate coding) is linear (or close to) in most empirical studies [25], [54], as well as in our HH simulations (see below). In theoretical studies, the concave 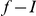 curve emerging at a homoclinic or Hopf bifurcation in the sole presence of action potential (AP) and leak currents [55] is linearized by adaptation and/or background synaptic noise [56], [57], [58]. This linear dependence writes:
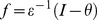
(1)where 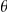 denotes the threshold current for spiking (rheobase) and 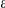 the inverse gain that we introduce for matters of simplicity (see below): 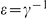, where 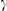 is the 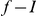 gain. Thus, by the chain rule, one has 
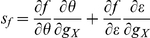
 so that after some algebra, on can write
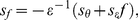
(2)where 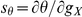 and 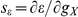, the threshold sensitivity and inverse gain sensitivity, measure how threshold and inverse gain vary upon 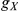 modifications. As shown in the next section, 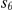 and 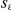 do not depend of the amount of conductance, 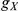, which implies that 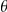 and 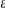 vary linearly with 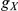, an unexpected finding given the strong non-linearity of HH systems. Rather, 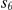 and 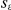 depend on the conductance kinetics. Hence, the effect of IP (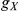 modification) on firing rate (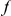) is the sum of two contributions that can be interpreted in contributions of the conductance kinetic properties.

On the one hand, 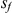 depends on the term 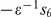 that is independent of firing frequency. Therefore, the causal mechanisms underlying this effect are expected to be mostly independent of AP occurrence. Good candidates include sub-threshold conductance significantly activated below the AP threshold, conductance with very smooth activation slope, or conductance with activation time-constants orders of magnitude larger than AP duration. Indeed, activation in these cases primarily depends on the mean inter-spike interval (ISI) membrane potential rather than on spike triggering. Because the term 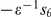 scales with threshold sensitivity, such conductance should display large 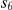 and plastic change thereof should thus affect the 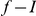 through threshold modifications.

On the other hand, 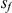 depends on the term 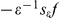 that scales with firing frequency. Hence, plastic conductance underlying this effect should display gating dynamics strongly correlated with spiking, e.g. implicated in spike triggering or triggered by the AP. Good candidates include conductance activating at depolarized sub-threshold potentials just beneath spike threshold or at supra-threshold potentials. Because this term 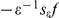 scales with the inverse gain sensitivity, such conductance should display large 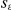 and their plastic modification should affect 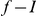 through gain modifications.

This initial analysis thus suggests that IP effects arise from two mechanisms underlain by conductance with distinct biophysical properties, according to whether they activate independent of, or correlative to spiking. Moreover, it suggests that such conductance should respectively exhibit strong threshold versus inverse gain sensitivities. In the following, we examine the biophysical plausibility of these suggestions by exploring the parameter space of the X conductance using extensive numerical simulations of HH neuron models (see Methods) to 1) unravel homogeneous regions of strong threshold or inverse gain sensitivities and 2) determine whether such regions indeed qualitatively correspond to the putative conductance regimes identified above. Furthermore, we 3) provide a theoretical analysis of integrate and fire (IAF) neuron models to investigate the underlying fundamental biophysical mechanisms, 4) evaluate the net frequency impact of threshold and gain modifications, 5) generalize our results to the IP of other conductance parameters and to several conductance and 6) illustrate how the present theory of IP effects can enlighten the outcome of IP rules, which would otherwise be unpredictable.

### Threshold and inverse gain linearly depend on 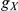


**Figure 1:**
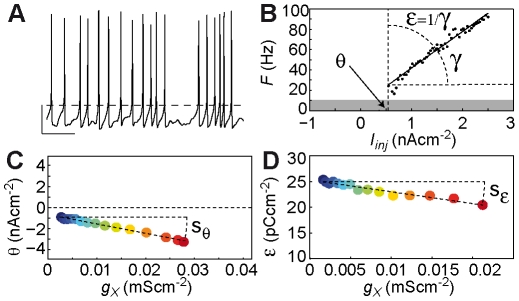
Computation of sensitivities. (**A**) Typical membrane potential trace in the standard HH model in response to a constant input current and random background synaptic current (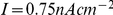, 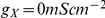). Scaling bars: 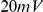, 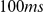. Dashed line: 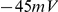 (**B**) Typical 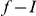 in the standard HH model. The current threshold for spiking, 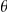 was defined as the first current eliciting a non null mean firing frequency. The inverse gain, 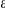, the inverse of the 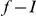 gain, 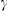, was estimated from linear regression (see Methods). (**C**) Estimate of 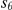, the threshold sensitivity, from the linear dependence of the threshold for spiking, 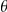, as a function of 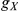 in the standard HH model (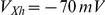 and 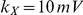; see Methods). (**D**) Estimate of 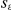, the inverse gain sensitivity, from the linear dependence of the inverse gain, 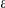, as a function of 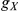 in the standard HH model (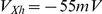 and 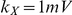; see Methods).

We ran extensive simulations of a single-compartment HH neuron model endowed with the X conductance submitted to a constant input current, 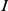, in addition to a random background synaptic current (see Methods; Figure 1A). We computed the mean firing frequency from individual simulations yielding 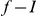 relations from which we computed 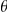 and 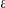 (Figure 1B; see Methods). As a general rule, both 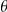 and 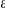 (Figure 1C–D) depend linearly on 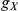:
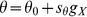
(3)and
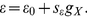
(4)


We found that this linear depedence holds for most of the parameter space, except for a limited parameter region (i.e. corresponding to small values of 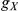 and of the e-fold activation slope 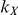, focalized half-activation potential to 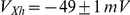, fast activation dynamics and reversal potential 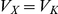) where limited amounts of non-linearity were observed. We thus computed 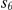 and 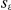 as linear regression slopes of 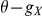 and 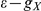 relations, respectively (Figure 1C–D; see Methods). Because of this linear dependence, we choose 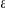 rather than 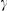 (which scales as 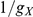). More importantly, because of linearity, 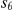 and 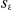 are constants independent of 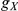, the amount of conductance present. Rather, they depend on the kinetic parameters and thus characterize how kinetic properties determine the effect of IP of 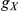 on the 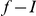.

### Threshold sensitivity in the standard HH model

**Figure 2:**
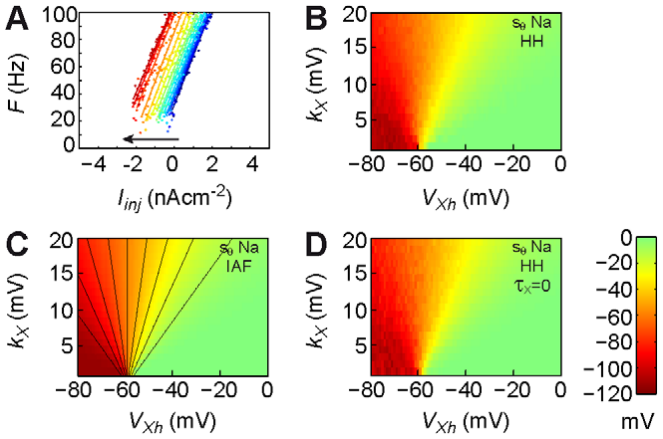
Threshold sensitivities of the standard HH model. (**A**) Increasing the maximal conductance 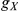 of the sodium conductance in the standard HH model with 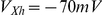 and 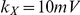 strongly shifts the 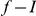 leftward (arrow), decreasing the threshold for firing, while leaving the inverse gain unchanged. Blue to red curves: 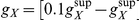 with 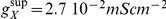 (see Methods for a definition of 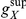). (**B**) Threshold sensitivity map of the standard HH model (sodium conductance), 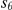 (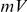) as a function of the half-activation potential (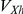) and e-fold slope of the Boltzmann activation voltage-dependence (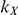). (**C**) Theoretical threshold sensitivity map derived from the IAF standard model (sodium conductance; 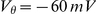). Black straight lines represent the isolines 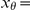
0.1 to 0.9 with 0.1 steps (see text). (**D**) Threshold sensitivity map of the standard HH model (sodium conductance) with instantaneous activation. (B, C) Colorbar as in (D).

We first examined 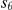, the threshold sensitivity, in a HH model endowed with an inward X conductance (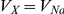) with time constant 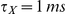, activation power 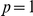 and no inactivation (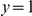). Hereafter, this model is termed the standard HH model. As expected, we found that increasing 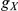 of this inward conductance lowered 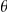 (Figure 2A; arrow), so that 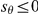. We systematically explored 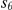 in the 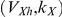 plane, building a 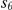 map to determine its dependence upon activation parameters (Figure 2B). The threshold sensitivity presents maximal absolute values for conductance steeply activating at hyperpolarized potentials (i.e. with most negative 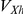 and small 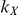), i.e. “sub-threshold” conductance. Conversely, 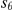 nearly vanishes for conductance steeply activating at depolarized potentials (with less negative 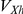 and small 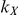), i.e. “supra-threshold” conductance. This result confirms our initial suggestion that conductance with large 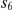 values activate relatively independently of firing frequency. Indeed, 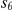 is the largest for most negative 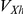 and small 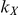, i.e. conductance fully activated at AP threshold, or with very large 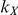, i.e. activation rather independent of membrane potential and thus of AP triggering (Figure 2B). We found that the global structure of the 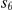 map in the standard HH model was robust to modifications in the parameter values, as well as to the specific type of the AP model (not shown). Moreover, this structure was generic across physiological ranges of activation kinetics and reversal potentials, as well as in the presence of inactivation (Text S2, S3, S4).

### A formal account of threshold sensitivity

To understand the dependence of 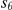 on parameters of the X conductance, we analyzed an IAF version of the standard HH model, the threshold IAF theory (see Methods and Text S1). We found that under the hypothesis of instantaneous activation, 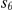 can analytically be approximated by
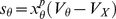
(5)where 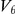 is the effective voltage AP threshold, 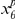 the steady-state activation at 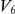 and p the activation power (Text S1). This expression provided an excellent match to the standard HH 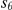 map (Figure 2C), as well as to the HH map obtained with instantaneous activation (Figure 2D), justifying our comparison between the HH standard model (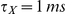) and the threshold IAF theory (instantaneous activation). Hence, equation (5) offers a direct interpretation of the radial structure of the 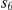 map: conductance with equal threshold sensitivities 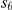 distribute along the iso–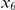 straight lines 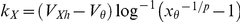 (Figure 2C). Their intersection point 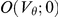 precisely matched the position of the central point in the standard HH model 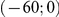. Together, these results reveal that IP threshold sensitivity is fundamentally accounted for by 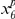, providing a precise criterion specifying the sub-/supra- threshold distinction emerging from our initial analysis. Moreover, we found that the threshold IAF theory generically accounted for the 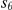 map across modifications throughout physiological ranges of activation kinetics and the reversal potential, as well as in the presence of inactivation (Text S2, S3, S4).

Together, these results strongly suggest that 1) large threshold sensitivity is characteristic of conductance with large activation at 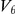 (the effective AP threshold) and 2) this is a generic property of voltage-gated conductance.

### Inverse gain sensitivity in the standard HH model

**Figure 3:**
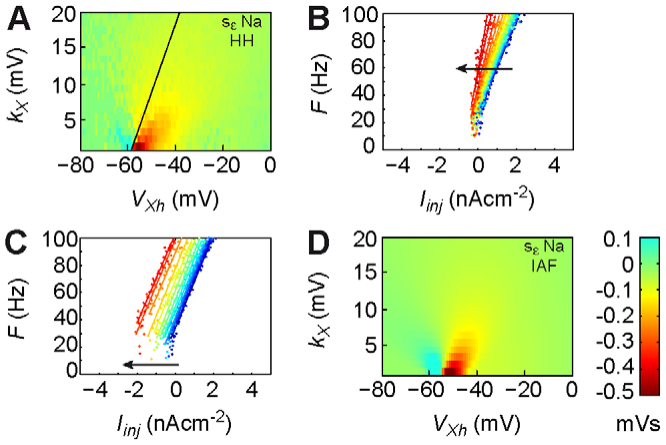
Inverse gain sensitivities of the standard HH model. (**A**) Inverse gain sensitivity map of the standard HH model, 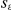 (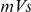). Black line: isoline 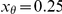. Colorbar as in (D). (**B**) Increasing the maximal conductance 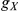 of the sodium conductance in the standard HH model with 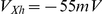 and 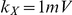 increases the gain (decreases the inverse gain) of the 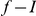 (arrow). Blue to red curves: 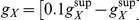 with 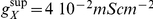. (**C**) Increasing the maximal conductance 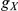 of the sodium conductance in the standard HH model with 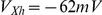 and 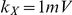 decreases the gain (increases the inverse gain) of the 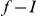 but this effect is masked by the much larger change of firing frequency due to modification of the threshold. Blue to red curves: 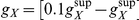 with 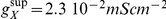. (**D**) Theoretical inverse gain sensitivity map derived from the pre/post-spike IAF theory with sodium conductance. 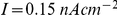, 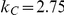.

We then examined the inverse gain sensitivity, 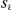, in the standard HH model. A parametric study in the 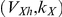 plane revealed a large domain of negative inverse gain sensitivities (Figure 3A). In this domain, increasing 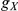 of the inward conductance (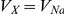) logically decreased the inverse gain (i.e. increased the gain) of the 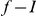 (Figure 3B; arrow). This domain essentially situated right to the isoline 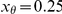 (Figure 3A; black line), corresponding to conductance with low threshold sensitivities (Figure 2B). It comprised an extended circular domain of moderate 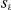, surrounding a restricted peak of large 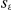 values focalized around O 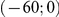. By contrast, conductance left to that isoline (i.e. with large threshold sensitivities, Figure 2B) displayed virtually null 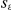. In a restricted zone of weak (paradoxical) positive 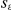 nearby the central point O (Figure 3A), a positive feedback with conductance activation rapidly brought the membrane potential to the threshold. This effect was most significant at long ISIs (small frequencies), increasing 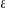. However, these frequency changes were minute compared to those arising from threshold modifications (Figure 3C) so we did not further study this effect.

The overall 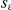 map structure was conserved with activation powers up to 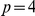, although it underwent a geometric distortion similar to that observed for the threshold sensitivity (Text S2). Thus, the property revealed generic that conductance with large 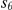 and those with large 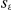 are located in disjoint domains of the parameter space, separated by the isoline 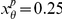. Specifically, large 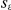 conductance activated at more depolarized potentials than large 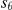 conductance, confirming our initial suggestion that conductance activating correlative to APs have large 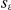 and low 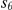.

### A formal account of inverse gain sensitivity

To unravel the mechanisms underlying inverse gain modifications, we assessed three IAF theory with specific activation dynamics correlated to spike occurrence. In the pre-spike IAF theory, where activation dynamics builds up before each forthcoming AP, the analytical 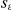 we have derived accounted for the peak of large 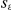 in Figure 3A (Text S6; Figure S5) and captured essential dynamical features of membrane potential and activation in that domain (Text S7; Figure S6). Complementarily, in the post-spike IAF theory, where activation dynamics relaxes following the AP, the analytical 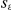 explained the large domain of moderate 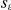 (Text S8 and S9; Figure S5) and the underlying dynamics (Text S10; Figure S7). However, these models did not account for 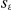 and the dynamics across the whole 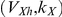 map.

To get a global account of 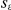, we devised the pre/post-spike IAF theory, which combines activation dynamics of the post-spike IAF theory during the initial part of the ISI with those of the pre-spike IAF theory for the end of the ISI (Text S11). We derived the analytical inverse gain sensitivity in this theory,
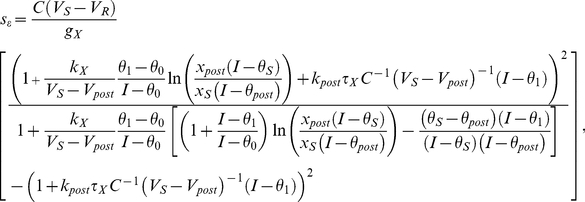
(6)where 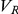 and 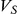 respectively denote the resting and phenomenological spike threshold potentials (Text S1), 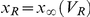, 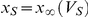, and 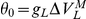, 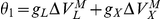, 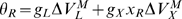 and 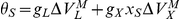 respectively denote the threshold current for activation 0, 1, 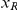 and 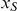, and where the post subscript refers to variables at the end of the initial period. This expression is more complex that those obtained from the pre- and post-spike IAF theories (Text S6 and S8), but it provided an excellent match to the map obtained from HH simulations, accounting both for the large domain of moderate 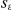 and the peak of large 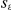 surrounding O (Figure 3D). Moreover, the pre/post-spike IAF theory captured the underlying neuronal dynamics with great accuracy (Text S12).

Together, these results suggest that conductance with AP-correlated activation display large 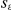 due to two combined mechanisms related to relaxation and buildup dynamics. Moreover, this property proved generic across physiological ranges of the reversal potential (even with the very different 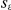 map structure characterizing potassium conductance; Text S13), activation kinetics (Text S14), as well as in the presence of inactivation (Text S15).

Globally, our study indicates a generic separation between domains of large threshold sensitivity and large inverse gain sensitivity. This generic character was further confirmed in two directions. First, we have shown that the dichotomy threshold versus inverse gain domains effectively translate into corresponding separate domains of net firing frequency changes (i.e. when considering physiological bounds of maximal conductance modifications; Text S16). Second, we have shown that the leak conductance, as well as the fast-inactivating sodium and potassium conductance of the AP, which undergo IP in real neurons, induce IP effects that are well accounted for by the general framework we have developed (Text S17).

### Generalization of IP to other parameters

Although maximal conductance is the most frequently modified parameter following induction protocols [28], [33], [35], [52], [53], IP can also regulate kinetic parameters, such as half-activation or half-inactivation potentials, e-fold slopes or time constants [59], [60], [61]. The theoretical framework we have developed can be extended to such forms of IP. Indeed, it can be easily shown from equations (3) and (4) that sensitivities of the threshold and inverse gain to a given parameter 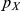 (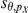 and 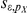) can be inferred from sensitivities to the maximal conductance 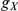:
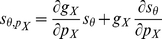
(7)and
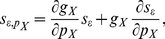
(8)where the first term of the RHS is zero whenever IP of 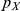 and 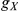 are independent or IP of 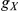 is absent (which does not preclude the calculation of 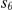 or 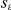). Therefore, the present results form a starting point from which it is possible to quantify the IP effect of kinetic parameters (either numerically, from 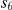 or 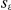 landscapes, or analytically from corresponding theoretical expressions). This is important, in particular because it may well allow unraveling essential regulatory subtleties of IP. Indeed, modifying the half-activation potential by a few mV, can, e.g., shift a plastic conductance between domains with totally different sensitivities. For instance, our study shows that a shift as small as 5–10 mV is sufficient to transform a plastic conductance that affects, for example, the 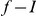 threshold into a conductance that affects its inverse gain. From this perspective, IP appears as a repertoire of plasticity mechanisms that includes its own forms of meta-plasticity.

### Understanding IP loop dynamics from IP effects

As emphasized in the Introduction, the present theory of IP effects is to be coupled with IP rules (i.e. activity-dependent biochemical regulations of conductance) to enlighten IP loop interactions. An exhaustive assessment of IP loops (e.g. homeostatic or anti-homeostatic regulation of large threshold or inverse gain sensitivity conductance) is well beyond the present scope and requires future studies. However, we provide here an illustrative case demonstrating how IP effects can enlighten the otherwise unpredictable outcome of IP rules. In this example, we assess how spontaneous discharge can emerge as a consequence of homeostatic IP (H/IP) in the absence of synaptic inputs. In the central nervous system, spontaneous activity is widespread [47], [48], [49] and strongly constraints both metabolic costs [51] and network dynamics [50]. Therefore, its regulation by H/IP is a strategic determinant of neuronal operations [45],[46]. Here, we evaluate 1) the conditions under which H/IP insures spontaneous discharge and 2) the extent to which the present theory of IP effects accounts for this property in the complex context of the IP loop.

To do so, we first study a HH model where the X conductance is regulated by a H/IP rule, implemented under the form of an aKP model ([43]; see Methods). This model was devised to account for the ubiquitous regulation of voltage-dependent membrane conductance by activity-dependent kinase and phosphatase cycles. In the aKP model, plastic changes arise from enzyme activation curves that monotonously translate neuronal activity, i.e. they are graded. This property is consistent with IP effects observed experimentally [32], [62], [63], [64], and departs from alternative bistable plasticity models in which autocatalysis induces binary switches [65], [66]. Moreover, in the aKP model, the time constant is activity-dependent, with slow dynamics at low activity and faster changes at higher levels. Again, this property accounts for experimental observation that homeostasy is rapid under conditions of hyper-activity [29], [33] and slower when activity is inhibited [35], [45]. Here, the maximal conductance, 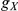, is set as the product between a total conductance and a functional fraction of channels conducing the X current. Spiking frequency is monotonously translated into intracellular calcium concentration dynamics via a high-threshold calcium conductance. In turn, calcium transients activate the kinase/phosphatase cycle, inducing plastic changes that decrease the functional fraction of the inward conductance upon increase in activity (see Methods). Thus, neuronal excitability opposes to activity changes.

**Figure 4:**
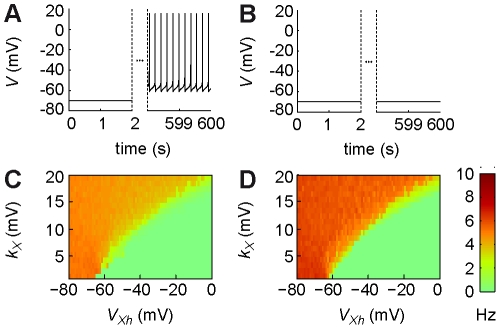
Spontaneous firing: inferring the outcome of the IP loop. (**A, B**) The emergence of a spontaneous discharge from H/IP depended from the conductance activation parameters ((A) 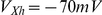, 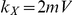; (B) ) 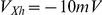, 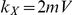;) in the HH-based loop model (see Methods). (**C**) Steady-state frequency of spontaneous firing in the 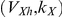 plane in the HH-based loop model. Colorbar as in (D). (**D**) Same as (C) in an equivalent rate-based model using IP effects (see Methods).

In the absence of synaptic drive, the neuron model is initially silent (Figure 4A) and intracellular calcium concentration rests at its basal level. Due to the homeostatic regulation, the functional fraction of the inward X conductance increases, thus increasing 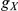 and neuronal excitability. This enhanced excitability primarily corresponds to a decrease in the current threshold, 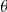, or an increase of the 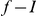 gain, 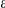, depending on activation parameters, because of the dichotomy unraveled in the present study. Because the neuron is initially silent, the input current is inferior to the threshold (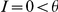). Therefore, increases in the 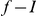 gain are ineffective. By contrast, decreases of the threshold eventually lead to the emergence of a spontaneous discharge. Thus, spontaneous spiking can naturally emerges (Figure 4A) or remain unexpressed, even at very large simulation times (Figure 4B), depending on whether the conductance regulated by the IP loop is sub-threshold or supra-threshold, respectively. Globally, spontaneous discharge indeed emerged only in the domain of large 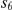 (Figure 4C), where the maximal possible threshold modification allowed firing at 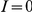 (note that the border superposed with the frontier of frequency changes arising from threshold modifications; Text S16). This border was robust to modifications of other parameters of the IP loop (e.g. kinetic parameters of the aKP model, the maximal calcium conductance), which essentially affected the overall spontaneous frequency level. Moreover, an IP loop model where HH equations were substituted for by equations (1), (3) and (5), i.e. rate coding with plastic threshold, yielded a similar map of spontaneous discharge (Figure 4D). Therefore, when coupled to the IP rule, the IAF theory properly accounted for the spontaneous discharge emerging in the presence of HH equations. Thus, HH equations can be replaced by IAF equations to unravel the outcome of the IP loop with lower computational cost and better tractability. Together, these results are consistent with the experimental observation that spontaneous discharge critically involves sub-threshold conductance [47], [48], [49] and that H/IP regulation of spontaneous firing operates through threshold modifications [45], [46].

These results illustrate how the dichotomy of IP effects we have unraveled qualitatively accounts for the outcome of IP, even within the context of the complex interactions that characterize the IP loop. Moreover, they illustrate how our theoretical description of IP effects is essential to quantitatively estimate the outcome of the IP rule, based on the precise knowledge of conductance biophysical parameters, which would be impossible otherwise. Therefore, the present theory, thanks to its concision, opens the way for a tractable analysis of the functional impact of the IP loop at the level of neural networks.

## Discussion

In the present study, we have achieved a theoretical and numerical sensitivity analysis aimed at systematically assessing the impact of voltage-gated maximal conductance modifications on the 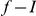. We focus on the 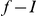 to explore the still largely obscure effects of IP on neuronal rate coding, which represents the functionally relevant regime of many neurons that share type I excitability, and is extensively employed experimentally to assess IP.

Our study leads to the general principle that the effect of maximal conductance plasticity on firing rate is governed by two additive terms which separately affect the threshold and the inverse gain of the 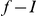. For a given maximal conductance change, these effects are weighted by two parameters, the threshold and the inverse gain sensitivities to the maximal conductance. These sensitivities are themselves independent of the maximal conductance. Rather, they reflect how kinetics (i.e. qualitative properties) of the conductance modulate the way maximal conductance changes affect the 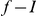. As anticipated by our initial theoretical analysis, an extensive exploration of sensitivities in the parameter space of HH models systematically demonstrated two contiguous, marginally overlapping domains of elevated threshold or inverse gain sensitivities in the 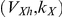 plane.

On the one hand, conductance activating “sub-threshold”, independent of the occurrence of spikes, display high threshold sensitivities, i.e. changes in their maximal conductance strongly affect the 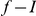 threshold of the neuron. Consistent, we have shown in analytically IAF neuron models that the threshold sensitivity solely depends on the activation level 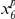 at 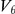, the effective AP threshold, which does not depend on firing frequency. Thus, IP in these cases affects the 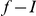 independently of firing frequency, i.e. it shifts the 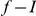 so that the threshold but not the gain is modified. Besides, it arises from the dependence of threshold sensibility on 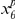 that conductance with very different activation functions but sharing the same 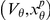 values present the same threshold sensitivity. By contrast, conductance with steep activation functions shifted by only a few mV can display extremely different threshold sensitivities. Thus, our results offer the possibility of estimating threshold sensitivity of real conductance assuming that their infinite activation and 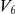 are known with reasonable accuracy.

On the other hand, conductance activating “supra-threshold”, i.e. in correlation with spikes, present high inverse gain sensitivities, i.e. changes in their maximal conductance strongly affect the 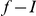 gain. We have shown that inverse gain sensitivity depends on two mechanisms related to the activation relaxation following APs, and the activation buildup preceding APs. Interestingly, these two mechanisms rely respectively on the difference between activation levels 1) attained at the end of the spike and at the reset potential and 2) the reset potential and the threshold potential. Hence, conductance displaying large inverse gain sensitivities have half-activation potentials situated above the reset potential and below ∼−20 mV and small e-fold slopes. Moreover, the pre-and post-spike IAF theories we have studied indicate that conductance with fast activation kinetics privilege the buildup effect while slower kinetics of activation favor the deactivation mechanism. As for the threshold sensitivity, we furthermore found that a precise knowledge of biophysical parameters can be crucial in estimating the inverse gain sensitivity of conductance.

Together, these results provide a unifying framework to account for and interpret IP experiments. Indeed, we have determined that voltage-gated conductance with large threshold versus large inverse gain sensitivities can be discriminated on the basis of a simple and generic criterion, i.e. an activation of 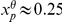. This criterion roughly corresponds to the classical albeit fuzzy distinction between “sub-threshold” and “supra-threshold” types of conductance. Hence, according to our results, pharmacologically identified conductance types such as the leak (I_L_), the persistent (I_NaP_) and slowly-inactivating (I_NaS_) sodium, the low-threshold calcium (I_CaT_), or the muscarinic (I_M_) and slowly-inactivating (I_Ks_) potassium conductance display biophysical parameters typically situated in the domain of high threshold sensitivity. Consistently, empirical studies indicate that modifications of the persistent sodium [67], slowly inactivating potassium [68] and leak conductance [69], [70] strongly correlate with large 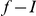 threshold modifications.

In contrast, modifications of pharmacologically identified conductance types such as the high-threshold calcium conductance (e.g. I_CaL_, I_CaR_), calcium-activated (I_AHP_) or fast-potassium potassium (I_A_) conductance that are directly or indirectly activated by APs should essentially affect the 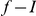 gain. Empirical evidence indicate that gain changes are indeed induced in vestibular nucleus neurons by IP of calcium-activated potassium conductance and spike triggered high-threshold R-type calcium conductance that induce their activation [54], [71]. Moreover, additional previous work has shown that the maximal conductance of calcium-activated current indeed determines the 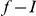 gain in proportion to their activation time constant [72], consistent with our analysis of the post-spike IAF theory.

The dichotomy we have unraveled and which appears to beneficiate from experimental support appeals several remarks. First, our results indicate that mixed modifications of the 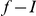 [25] do not necessarily implicate the co-regulation of two or more conductance but could simply arise from the IP of conductance situated at the overlapping of threshold and inverse gain domains. Second, parallel to the sub-/supra-threshold dichotomy, our study clearly indicates the opposition between sodium and potassium conductance of the AP, which respectively affect the threshold versus the inverse gain of the 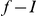, consistent with experimental data [60], [73]. Third, experimental data indicate that the IP of several A-type or persistent potassium conductance affects the 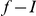 threshold [29], [74], while these conductance are paradoxically traditionally classified as supra-threshold because they present quite depolarized half-activation potentials (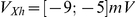). However, these conductance present large e-fold activation slopes (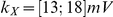) so they should lie in the domain of large threshold modifications. Thus, based on actual biophysical conductance parameters, the present theory correctly categorizes the IP effects of pharmacologically identified conductance, even when their apparent classification, based on the fuzzy sub/supra-threshold distinction, is misleading. Our study therefore points toward the importance of precise biophysical conductance parameters over the simple knowledge of the pharmacological conductance type in determining the rate effects of the IP of actual conductance. Finally, the validity of our theoretical results could practically be further confirmed or infirmed in detail, employing the dynamic clamp technique to experimentally measure threshold and inverse sensitivities by sampling points of interest in the biophysical parameter space. In particular, this technique could help disentangle an apparent discrepancy that we have unraveled concerning the I_H_ conductance. Indeed, our results predict no effect on the gain and a negative 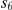 because I_H_ is depolarizing (not shown), whereas several IP studies show that I_H_ exclusively increases the 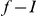 threshold [29], [33], [53]. This may originate from indirect effects such as a decreased input resistance or putative complex interactions with other sub-threshold currents [75] and geometrical factors in dendrites [76]. The dynamic-clamp technique may thus separate direct and indirect effects in that case.

In addition to interpreting existing results, the present theoretical framework represents a valuable tool for experimentalists to target putative conductance involved in IP, based on the observation of 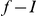 changes. Moreover, our analysis has unraveled supplementary intermediate electrophysiological observables such the effective AP threshold (Text S1) or the ISI voltage trajectory (Text S7, S9, S10), which modifications can be analyzed to refine the targeting of putative conductance of interest.

We have ascertained that the present results are robust. Indeed, shifting half-activation potentials of AP sodium and potassium currents by a few mV shifts sensitivity maps by the same amount along the 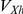 dimension but does not change their global structure (not shown). Moreover, using another model of AP conductance did not significantly change our results (not shown; [77]). Furthermore, the threshold versus inverse gain sensitivity dichotomy we have demonstrated proved robust when considering net mean frequency effects that can be obtained from maximal modifications of maximal conductance preserving excitability parameters within physiological bounds (Text S16).

Besides, the dichotomy we have unraveled appears to extend to the general case of voltage-dependent activation time constants, commonly encountered in real conductance. Hence, sensitivity maps obtained with voltage–dependence activation time constants (in the range 1–5 ms; not shown) were consistent with our previous understanding of sensitivities' dependence on time constants. Indeed, we found (not shown) that 1) threshold sensitivity is globally unaffected by the voltage-dependence of the activation; 2) large time constants at ISI potentials (below voltage AP threshold) increase the impact of the post-spike relaxation and delay the build-up effect, thus augmenting the post-spike mechanism and diminishing the pre-spike mechanism; 3) large time constants at spike potentials (above AP voltage threshold) diminish the activation increase during the spike, reducing the post-spike effect, but have no impact on the pre-spike mechanism.

Finally, an important question is whether the IP effects we unravel are robust in the general case where several voltage-gated conductance are present, even though exploring this issue in depth is largely out of the present scope. Actually, we have achieved a preliminary exploration suggesting that threshold and inverse gain modifications behave as the linear sum of individual conductance effects. If confirmed, this result would be noteworthy, given the degree of non-linearity commonly encountered in neurons at the level of the membrane potential or gating variables. Moreover, such linearity would open the possibility to capture complex interactions between conductance in a simple way in terms of frequency coding in neuron and neural network models.

Although robust, our results should be extended with respect to several dimensions, including 1) IP effects on spike-timing properties (e.g. higher order moments of the discharge, resonance, latency to first spike or frequency adaptation), in particular by also considering type II excitability neuron models, 2) multi-compartmental neuron models to address IP effects on dendritic integration [63], summation [78], branch computation [79] and spike back-propagation [80] and determine whether the sub-/supra-threshold distinction remains relevant with dendritic spikes.

This analysis complements recent analyses of parameter robustness of excitability in Hodgkin-Huxley (HH) models, using sensitivity analysis or stochastic search methods [81], [82], [83], [84]. Indeed, these studies assess the spontaneous dynamical regime of neurons [82], [83] or incomplete descriptions of the excitability [81], [84], whereas our study fully quantifies the 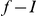. Moreover, they seek compensatory trade-off between conductance with specific kinetics, in the space of maximal conductance dimensions. Rather, our study is independent of the rules that actually govern IP (e.g. H/IP versus AH/IP) and explores the kinetics parameter space of a single generic model of voltage-gated conductance. Therefore, it allows evaluating independently the sensitivity of virtually any voltage-gated conductance with arbitrary kinetics and offers some insights on calcium- or second-messenger gated conductance scaling with firing frequency [85], [86], [87].

Here, we have focused on IP effects to escape the entanglement of IP effects and IP rules in empirical and theoretical studies and provide a manageable framework for the comprehensive study of IP loops. Hence, our goal is attainable by coupling the present IP effect equations with IP rules equations describing the causal mechanisms relating on-going spiking activity to conductance changes. In our mind, realistic signaling pathways models are desirable as they share the same - molecular - level of description. IP processes display gradation [54], [71], [88], [89], possibly fast induction [62], [90], long-term maintenance [8], [71], [91] and ubiquitously involve kinase/phosphatase cycles [21], [60], [89] so that the aKP model [43] represents a natural counterpart to the present model. In the present study, we have coupled the aKP model to HH or rate coding equations to address the example of the homeostatic regulation of spontaneous discharge by the IP loop. Our results illustrate how the IP theory we have unraveled can account for the outcome of IP rules, based on the precise knowledge of conductance biophysical parameters, and provide lower computational cost and better tractability to systematically decipher the complexity of the IP loop. To model the loop, choosing autocatalytic plasticity models inducing binary switches of the plastic variable would have been clearly irrelevant [65], [66], because homeostatic IP changes are graded [54], [71], [88], [89]. Similar results would be obtained using an alternative phenomenological model that produce graded changes [38]. However, because it lacks activity-dependent time constant, such a model would fail - contrarily to the aKP model - to account for the slower dynamics at low electrical activity [35], [45] and faster changes under conditions of hyper-activity [29], [33] that characterize homeostatic IP experimentally. In the future, one may in a similar way realistically investigate essential issues related to IP loops at the single neuron level such as the stability problem, the emergence of dynamics of interest, information processing properties or interactions with synaptic plasticity.

Introducing these coupled equations in neural networks offers the possibility to assess the impact of IP on dynamical and computational network properties. The present results allow studying IP of real conductance with known biophysical parameters in firing rate neural networks with explicit threshold and/or gain, and spiking neural networks embedded with conductance parameters, using event-based schemes [92] by taking advantage of the analytical voltage trajectories we have devised. Studying such networks would allow assessing the causal role of conductance modifications that have been correlated to various behavioral learning (e.g. trace, classical and operant conditioning, or rule learning; [6], [7], [93]. They would also bring about gaining a global picture of the computational properties conferred by IP. Indeed, modifying the 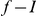 threshold provides an additive modulation determining input selectivity, while 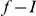 gain modifications operate a multiplicative modulation that scales neuronal output. These distinct forms of activity-dependent regulations should therefore participate setting very different computational properties at the level of neural networks [94] with regard to dynamical regime control, information storage or history-dependent computations for instance.

As a concluding remark, the present results are independent of the regulatory processes modifying conductance parameters and thus relevant to a larger class of processes than IP, possibly including neural development [95], maturation [2], neuromodulation [96], aging [96] and various neural diseases [13], in which conductance modifications represent critical cellular processes.

## Methods

### General principles

The present study is aimed at providing a description of the effects of conductance parameter modification on neuronal excitability. Characterizing neuronal excitability requires criteria to evaluate activity in response to stimulatory inputs. Here, we do not study possible changes in dynamical regimes of firing (e.g. regular spiking, intrinsic bursting) that can result from IP regulation [89]. Rather, we focus on neuronal rate coding, which represents the functional regime of type I excitability neurons and a central determinant of asynchronous activity in the awaken state in cardinal central structures.

Spiking rate and spike timing represent two complementary dimensions by which information can be carried by neurons in a regular spiking mode and that can be analyzed to this end [97], [98]. In the present study, we considered the frequency-current intensity (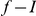) relation that is widely employed to characterize firing rate before and after IP induction in empirical studies. Actually, most in vitro IP studies demonstrate activity-dependent changes of the 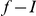, some of which provide information about underlying conductance changes, allowing to test modeling predictions. By contrast, changes in spike patterns [88], [89], [99] following IP protocols appear very diverse, so that getting a consistent view of these effects remains speculative.

Reaching present conclusions required evaluating the response of the standard model in >10^7^ conditions: we computed >10 maps (see Parametric exploration), each map incorporated >10^3^ points at which sensitivities were computed from ∼30 simulated 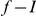, with each 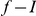 from ∼30 injected current values. The dynamic-clamp method allows evaluating at best a few hundred conditions in real neurons [100], i.e. an exploration capability that is three order of magnitude lower. Thus, such an exploration is practically impossible to achieve, even in vitro, because of the short lifetime of experimental preparations, justifying the use of numerical simulations and the search for analytical descriptions of the IP effects.

### Hodgkin-Huxley models

Here, we study a single-compartment neuron model endowed with leak, AP currents and a generic voltage-dependent X current to assess the effect of its plasticity on firing rate of the model neuron. The membrane potential (V) evolves according to
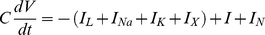
(9)where 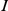 is the input current and 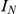 is a background noise synaptic current (see Noise currents). The leak current can be written as
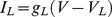
(10)and AP currents are taken from a model of excitatory regular-spiking neocortical cells [101]. The X current follows
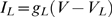
(11)where 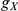 is the maximal conductance, 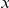 and 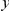 respectively denote activation and inactivation variables, 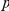 is the activation power and 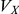 the reversal potential. Activation x follows first-order kinetics
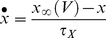
(12)with time-constant 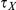 and
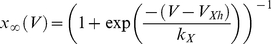
(13)where 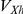 and 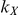 respectively denote the half-activation potential and e-fold slope of the Boltzmann activation voltage-dependence. The inactivation variable 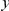 is described using the same formalism, with parameters 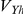, 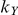 and 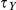.

The kinetics formalism we employ offers two decisive advantages to reach the present goal of unraveling the effect of IP on firing rate. First, this formalism is simple enough to derive tractable analytic expressions of 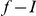 relations and sensitivities, which dependence on biophysical parameters is essential to summarize the impact of voltage-dependent conductance IP on firing rate. Second, this formalism is sufficiently rich to offer a common framework to describe voltage-dependent membrane conductance, as it is commonly used to fit experimental data with excellent qualitative and quantitative match. In the following, we first consider the case where inactivation is lacking (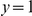) as a starting, analytically tractable scenario. This scheme encompasses the case of sub–threshold persistent Na (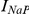) currents, muscarinic potassium (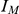) currents, inwardly-rectifying cation (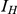) currents, or non-inactivating supra–threshold calcium (e.g. 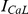) currents. We also address the case of inactivating conductance, encompassing slowly inactivating sodium currents (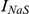), fast-inactivating (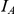) and slow-inactivating (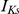) potassium currents, as well as low-threshold calcium currents (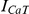). Note that the case of calcium- or second-messenger-activated (e.g. BK or SK potassium) conductance is not addressed by this study. However, our conclusions allow deriving strong inference on the IP of these conductance. Therefore, this formalism offers a single common parameter space wherein it is possible to systematically explore the plasticity impact of cardinal voltage-gated conductance.

### Parametric exploration

Assessing the effect of plastic conductance modifications on firing rate requires in principle the exploration of a 9-dimensional space with the present formalism, the X conductance being described by the parameter vector 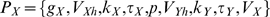. However, we organized the exploration in a hierarchical manner for the following reasons. The maximal conductance 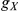 was considered a priori as the primary parameter because it is the most commonly modified biophysical parameter in IP empirical studies [28], [33], [35], [52], [53]. The sensitivity analysis we achieved confirmed the pertinence of this choice a posteriori, because computing 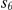 and 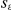 (see below) fully captures the effect of 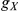 on firing rate, removing this dimension from the exploration of the parameter space. Besides, the theoretical analysis opening the Results leads to the suggestion that the conductance voltage-dependence is likely to be an important determinant of IP effects on firing rate (see Results). Thus, we conducted sensitivity map analysis in the 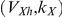 space to unravel these effects (see Sensitivity map analysis). These parameters generally distribute within 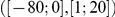 for most sodium, calcium and potassium conductance so we restricted our map analysis to these ranges with 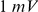 resolution along both dimensions. Such maps were built for a few representative values of kinetic order and time constant to preserve map representation for the sake of comparison (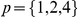 and 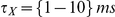). For clarity, we did not thoroughly explore the inactivation parameter space but rather illustrated some typical examples. Hence, we used a conservative set of voltage-dependency parameters 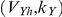 that is representative of actual inactivation experimental data (see Standard parameters). With respect to inactivation dynamics, we computed sensitivity maps for representative values of the inactivation time constant (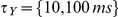). Finally, we explored the effect of typical sodium, calcium, and potassium conductance values of the reversal potential, 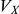 (see Standard Parameters). Note that time constants do not depend on voltage in our model, whereas they usually do in real neurons. Considering voltage-dependence of time constant would bring several additional parameters and there is no canonical framework describing these dependencies (e.g. exponential, sigmoid, bell-shaped). Thus, considering the whole complexity of kinetic time constants would expand the scope of the study disproportionately.

### Sensitivity map analysis

To estimate the threshold (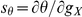) and inverse gain (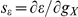) sensitivities at each point of the 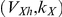 grid, we first computed the current threshold 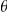 and inverse gain 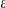 by linear regression of the 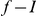 for different 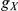 values at that 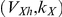 point. The 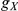 values geometrically spanned the range 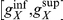 with reason 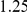 to explore the several order of magnitudes that usually separate 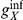 and 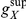 in an algorithmically efficient way; 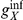 and 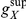 were determined as following.

Because of the geometrical progression, 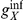 had to be chosen non-null. In the absence of any a priori knowledge on the magnitude at which an X conductance with parameters 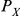 begins affecting 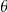 or 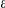 significantly, we empirically chose 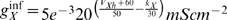. This formula insured no significant modification of 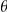 or 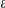 (i.e. compared to the model without the X conductance), while allowing reasonable computing time to reach 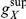 (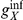 was about three orders of magnitude below 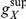). We checked that the exact value of 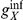 had no impact on the present results.


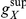
 was defined as the maximal 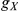 value of the geometrical progression for which the model did not exhibit one of the following categories of undesirable properties: 1) unrealistic large 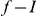 threshold, 2) unrealistic large negative 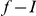 threshold, 3) unrealistic small 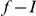 gain, 4) unrealistic large 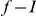 gain, 5) saturation, 6) unrealistic short AP duration, 7) unrealistic long AP duration. Neurons were considered of category 1) when no discharge was elicited for an injected current of 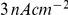 and of category 2) whenever they spontaneously discharge at 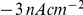. 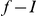 gain bounds were taken 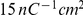 and 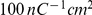, i.e. 1/2.5 and 2.5 times the 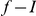 gain in the absence of the X conductance, 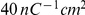, consistent with typical bounds found in vivo in cortical pyramidal neurons [25]. Similarly, we confined the AP duration within 1.8 ms +/−25%.

Both the threshold and inverse gain almost systematically linearly depended on 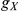 (see Threshold and inverse gain linearly depend on 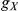, in Results). We thus computed 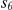 and 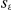 sensitivities as the linear regression slope of the 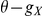 and 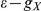 relations, respectively (Figure 1C–D).

### Threshold and inverse gain computation of the 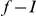


For each 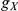 within the range 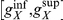, a current threshold and an inverse gain were estimated from 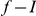 relations obtained through HH simulations. The 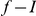 relation has no a priori reason to be strictly linear as it arises from the complex interactions between the theoretical non-linear 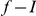 function of type I excitability [55] and the additional influences of noise and the generic X conductance. However, as in numerous other theoretical or empirical studies (e.g. [25]), we found the 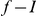 to be very close to linearity in practice, as we systematically observed extremely strong statistical significance of the correlation coefficient test (i.e. typically 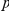-values in the range 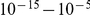; see below). The mean spiking frequency was measured at 30 different intensities uniformly distributed in the range 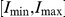, where 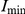 and 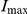 were defined as the currents respectively eliciting 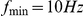 and 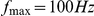. 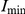 was chosen to cut out the lower part of the 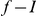 which is dominated by the complex interaction between the theoretical infinite slope at limit cycle bifurcation and the linearizing effect of background noise. 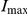 was chosen to avoid frequency saturation effects. Together, defining these bounds allowed estimating threshold and inverse gain based on the linear part of the 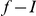. For each tested intensity, the mean frequency was computed over 30 spikes (i.e. ISI intervals), with initial conditions for conductance activation and inactivation variables taken at their steady-states values, in order to avoid the effects of slow dynamics. The simplest way to characterize the 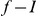 as a linear dependence, 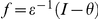, is to estimate 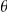, the current threshold and 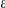, the inverse gain, by linear regression of current/frequency couples. Between 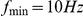 and 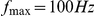, this procedure gave an excellent estimate of the nearly perfectly linear relation observed from HH simulations. However, because the frequencies used to compute the linear regression are superior to 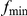 (i.e. non null), systematic bias on the estimation of the threshold 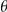 could arise in domains of the parameter space where 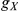 modifications induced large changes in the 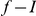 gain. To avoid that bias, we simply determined the current threshold for spiking as the first current eliciting a non-null mean firing frequency. If we denote that estimation 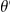, one has 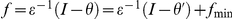. The theoretical frequency sensitivity relation we establish (equation (2)), as well as the net frequency variation relation derived from it (equation (16.4), Text S16) are based on derivation with respect to the maximal conductance 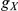. Because 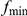 is a constant independent of 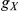, it disappears by derivation so that these relations remain unchanged using 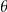 or 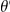. Thus, for the sake of simplicity, we use 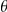 in equation (1), but technically compute 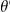 throughout results. Note that in IAF theories, the values of 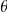 differ from that estimated from HH simulations because there is no noise current bias added (in addition to model simplifications). However, we get excellent estimation of threshold sensitivities because they do not rely on the exact value of threshold but on relative variations due to 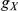 modifications. Note also that threshold and inverse gain sensitivity is independent of 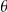 in HH simulations, by definition. Finally, note that the 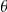, 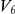 and 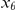 variables appearing in inverse gain sensitivity expressions in IAF theories rely on a distinct definition of the threshold in terms of voltage that is independent on whether one chooses 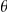 or 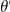 from the 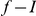 in HH simulations.

### Noise currents

Random excitatory and inhibitory synaptic currents were injected to HH models to represent the background synaptic tonic influence exerted in vivo that induces spontaneous discharge and linearization of the 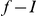. The synaptic currents were modeled as in [102]. For excitatory conductance, we considered 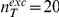 independent trains that each comprised 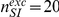 synchronized inputs firing at 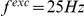. The unitary conductance of each input was 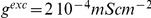 and its time-constant 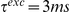. For inhibitory conductance, we used 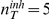, 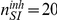, 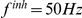, 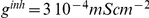 and 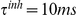. These parameters provided a net excitatory current yielding a mean spontaneous firing frequency of 15 Hz. The results we obtain were robust to the exact quantitative details of the synaptic drive.

### Integrate-and-fire theory

In integrate-and-fire (IAF) models, the objective was to reach analytical expressions of the threshold (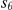) and inverse gain (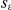) sensitivities that could capture the mechanisms underlying IP impact on firing rate. Computing these sensitivities in turn requires getting formal descriptions of the 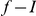. The relation between ISI duration (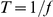) and the input current (I) can be achieved by formally integrating (when possible) the 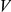-differential equation from the reset potential (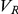) to the onset AP threshold (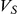). Because equation (12) is coupled to several non-linear differential equations governing the evolution of AP and X currents' gating variables, it cannot be integrated analytically without further simplifications. As a first simplification, AP currents are eliminated, as their activation variables are negligible during the ISI. This approximation is reasonable for frequencies up to a hundred Hz, as the slowest time constant of these currents is ∼3 ms. The voltage derivative then can be written as
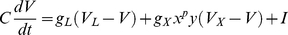
(14)Under further simplifying hypotheses, activation and inactivation of the X conductance express as explicit functions of time and the membrane potential (i.e. 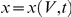, 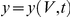) so that equation (17) can possibly be integrated to reach analytical expressions of 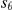 and 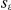 (Text S1, S4, S6, S8, S9, S11, S14, S15). Numeric evaluation of the expressions obtained with IAF theories depend on the value of 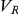 and 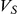. These values were globally constant across the different parameter sets tested in the present study and usually vary a few mV around their mean value (see Standard parameters). In IAF theories, 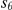 was computed at each 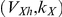 point of the map using 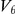 and 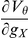 averages over 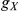, with 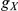 geometrically spanning 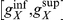 with reason 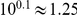. 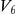 values were numerically computed as the potential corresponding to the minimum of the 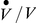 relation for each parameter set and 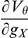 was numerically estimated as 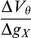 from 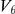 values obtained with successive 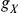 values. In the different IAF theories, we compute the frequency as the inverse of the inter-spike interval (ISI) duration: 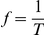 for the sake of simplicity. Actually, correctly computing the firing frequency would imply computing 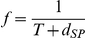, where 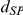 is the spike duration, but it would render calculations cumbersome. In doing so, we produce an error 
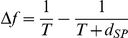
 that increases with frequency and produce a bias on estimation of the inverse gain 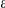. However, the mean value of this error can be written as 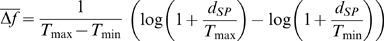, with 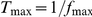 and 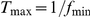, so that 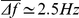, i.e. it is minute. Consistent with this, IAF theories accounted nicely both quantitatively with membrane potential and X activation dynamics, as well as with sensitivity maps obtained from HH simulations.

### Modeling the IP loop

The IP loop model is derived from the HH standard model, in which the X conductance is regulated by a H/IP rule. The membrane potential evolves according to
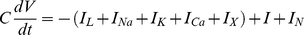
(15)where the additional high-threshold calcium current
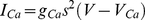
(16)is introduced to monotonously translate spiking frequency into intracellular calcium concentration dynamics. All other currents were set as described above. The activation variable 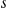 follows first-order kinetics
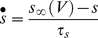
(17)with time-constant 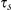 and
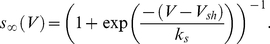
(18)Calcium concentration dynamics is modeled as resulting from the inward influx due to 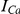 and first-order buffering:
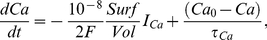
(19)where 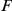 is the Faraday constant, 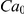 is the basal intracellular calcium concentration, 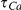 is the buffering time constant, and
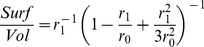
(20)surface area to volume ratio of an idealized intracellular shell compartment of thickness 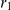 situated beneath the surface of a spherical neuron soma of radius 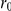.

Following the formalism of the aKP model (see [43]), the intracellular calcium concentration activates a kinase/phosphatase cycle that determines the phosphorylated fraction 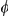 of the X conductance, according to
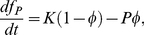
(21)where the macroscopic reaction rates of the kinase and phosphatase enzymes are
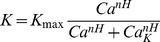
(22)and
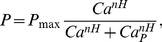
(23)with 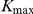 and 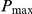 being the maximal reaction rates, 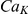 and 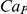 the half-activation calcium concentrations, and 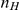 the Hille number of enzymatic reactions.

The maximal conductance is set as
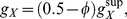
(24)where 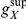 is the superior physiological value of the X conductance corresponding to the 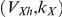 parameter considered (Text, S16). Thus, strong spiking activity translates as large 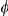 values, yields low 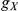 values of the inward conductance and decreases excitability, i.e. regulation is homeostatic.

In a rate-coding version of the IP loop model, the firing frequency of the model followed
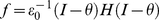
(25)where 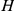 is the Heaviside function, 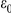 is the basal value of the inverse gain. The threshold evolved according the threshold IAF theory (equation (1.2), Text S1), in which the maximal conductance was plastic (equation (24)):
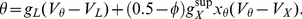
(26)The intracellular calcium concentration was set as
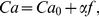
(27)where 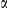 was fitted from the nearly linear calcium-spiking frequency relation in HH models.

### Statistical and numerical procedures

All linear regression analysis of the present study (i.e. 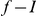, 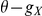, 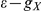) were of the model I-type. The statistical significance of the regression was assessed with the test of the correlation coefficient with p-value<0.05, statistic 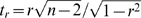 and 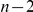 degrees of freedom, where 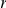 is the coefficient of regression and 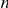 the number of observations. The models were numerically integrated using Runge-Kutta 4^th^ order integration.

### Standard parameters

Unless stated, the standard parameter values we use for the X conductance are 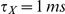, 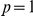, 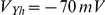, 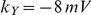, 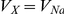. AP current parameters are those of [101]. Leak current parameters are 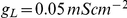 and 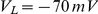. Reversal potentials were taken as 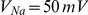, 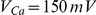, 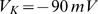. In IAF theories, 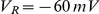, 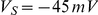, 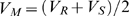, 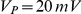, 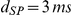. In the IP loop model, 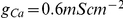, 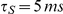, 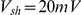, 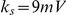, 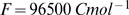, 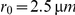, 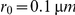, 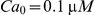, 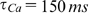, 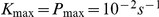, 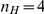, 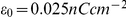, 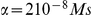.

## Supporting Information

Figure S1Threshold sensitivity and activation kinetics. (A) Theoretical threshold sensitivity map derived from the threshold IAF theory computed from equation (1.3). (B) Threshold sensitivity map of the standard HH model with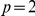. (C) Threshold sensitivity map of the standard HH model with 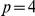. (D) Theoretical threshold sensitivity map derived from the threshold IAF theory with 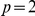. Black line: isocline 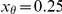. (E) Theoretical threshold sensitivity map derived from the threshold IAF theory with 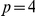. Black line: isocline 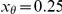. (F) Activation curves of three conductance with very different 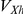, 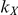
and 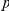 share the same activation at 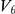, 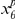 and thus the same threshold sensitivity 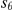 (black dot). Solid line: 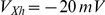, 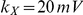, 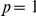; dotted line: 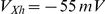, 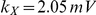, 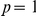; dashed line: 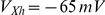, 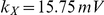, 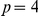. (G) Threshold sensitivity map of the standard HH model (sodium conductance) with activation time constant 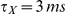. (H) Same as (G), with 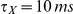. (A, B, C, E, G) Colorbar as in (H).(TIF)

Figure S2Threshold sensitivity and reversal potential. (A) Threshold sensitivity map of the standard HH model with potassium currents (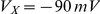). Colorbar as in (B). (B) Theoretical threshold sensitivity map derived of the threshold IAF theory with potassium conductance (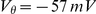). (C) Threshold sensitivity map of the standard HH model with calcium conductance (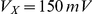). Colorbar as in (D). (D) Theoretical threshold sensitivity map derived from the threshold IAF theory with calcium conductance (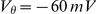).(TIF)

Figure S3Threshold sensitivity and inactivation. (A) Threshold sensitivity map of the standard HH model in the presence of inactivation (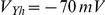, 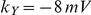, 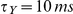). Colorbar as in (B). (B) Theoretical threshold sensitivity map derived from the threshold IAF theory with inactivation.(TIF)

Figure S4Inverse gain sensitivity and activation power. (A) Inverse efficacy sensitivity map of the standard HH model, 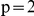. Black line: isocline 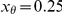. Colorbar as in (B). (B) Inverse efficacy sensitivity map of the standard HH model, 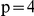. Black line: isocline 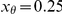.(TIF)

Figure S5 Inverse gain sensitivity in the pre- and post-spike IAF theories. (A) Theoretical inverse gain sensitivity map derived from the post-spike IAF theory with sodium conductance. (B) Theoretical inverse gain sensitivity map derived from the pre-spike IAF theory with sodium conductance.
(TIF)

Figure S6ISI dynamics for a sodium X conductance in the domain of large inverse gain sensitivities. (A) Mean membrane potential dynamics of the standard HH model for increasing 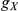 with adjusted input currents to match a common firing frequency of 50 Hz. Blue to red curves: 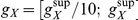 with 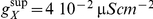 (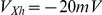 and 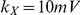). (B) X conductance activation dynamics corresponding to (A). (C) Theoretical membrane potential dynamics in the pre-spike IAF theory, with 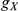, 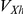 and 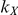 similar to (A). (D) Activation dynamics corresponding to (C).(TIF)

Figure S7ISI dynamics for a sodium X conductance in the large domain of moderate inverse gain sensitivities. (A) Mean membrane potential dynamics of the standard HH model for increasing 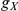 with adjusted input currents to match a common firing frequency of 50 Hz. Blue to red curves: 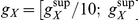 with 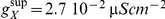 (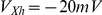 and 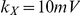). (B) X conductance activation dynamics corresponding to (A). (C) Theoretical membrane potential dynamics in the post-spike IAF theory, with 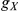, 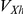 and 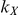 similar to (A). (D) Activation dynamics corresponding to (C).(TIF)

Figure S8 ISI dynamics for a sodium X conductance in the pre/post-spike theory. (A) Theoretical membrane potential dynamics in the pre/post-spike IAF theory, with parameters similar to those used in Figure S6. (B) Activation dynamics corresponding to (A). (C) Theoretical membrane potential dynamics in the pre/post-spike IAF theory, with parameters similar to those used in Figure S7. (D) Activation dynamics corresponding to (C).
(TIF)

Figure S9ISI dynamics for a potassium X conductance in the pre/post-spike theory. (A) Mean membrane potential dynamics of the standard HH model with potassium conductance, for increasing 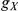 with adjusted input currents to match a common firing frequency of 50 Hz. Blue to red curves: 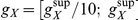 with 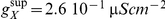 (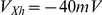 and 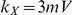). (B) X conductance activation dynamics corresponding to (A). (C) Theoretical membrane potential dynamics in the pre/post-spike IAF theory, with 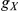, 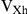 and 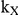 similar to (A). (D) Activation dynamics corresponding to (C).(TIF)

Figure S10Inverse gain sensitivity and reversal potential. (A) Inverse gain sensitivity map of the standard HH model with calcium conductance. (B) Theoretical inverse gain sensitivity map derived from the pre/post-spike IAF theory with calcium conductance. (C) Inverse gain sensitivity map of the HH model, with potassium conductance. Colorbar as in (D). (D) Theoretical inverse gain sensitivity map derived from the pre/post-spike IAF theory with potassium conductance. 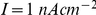, 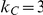.(TIF)

Figure S11Inverse gain sensitivity and activation time constant. (A) Inverse gain sensitivity map of the standard HH model with instantaneous activation. Colorbar as in (B). (B) Inverse gain sensitivity map of the standard HH model with sodium conductance with activation time constant 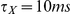.(TIF)

Figure S12Inverse gain sensitivities and inactivation. (A) Inverse gain sensitivity map of the standard HH model in the presence of inactivation (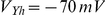, 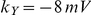, 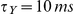). Colorbar as in (B). (B) Theoretical inverse gain sensitivity map derived from the pre/post IAF theory with inactivation.(TIF)

Figure S13 Net frequency effects arising from threshold and inverse gain modulation. (A) Map of the common logarithm of maximal conductance limit for sodium conductance. Colorbar as in (B). (B) Map of the common logarithm of maximal conductance limit for potassium conductance. (C) Net frequency effects from threshold modulation for sodium conductance. Colorbar as in (D). (D) Net frequency effects from inverse gain modulation for sodium conductance. (E) Net frequency effects from threshold modulation for potassium conductance. Colorbar as in (F). (F) Net frequency effects from inverse gain modulation for potassium conductance.
(TIF)

Text S1 The threshold IAF theory
(DOC)

Text S2 Threshold sensitivity and activation kinetics
(DOC)

Text S3 Threshold sensitivity and reversal potential
(DOC)

Text S4 Threshold sensitivity and inactivation
(DOC)

Text S5 Inverse gain sensitivity and activation power
(DOC)

Text S6 The pre-spike IAF theory
(DOC)

Text S7 Comparison of dynamics in the standard HH and pre-spike IAF theory
(DOC)

Text S8 The post-spike IAF theory
(DOC)

Text S9 Activation dynamics during the spike
(DOC)

Text S10 Comparison of dynamics in the standard HH and post-spike IAF theory
(DOC)

Text S11 The pre/post-spike IAF theory
(DOC)

Text S12 Comparison of dynamics in the standard HH and pre/post-spike IAF theory
(DOC)

Text S13 Inverse gain sensitivity and reversal potential
(DOC)

Text S14 Inverse gain sensitivity and activation time-constant
(DOC)

Text S15 Inverse gain sensitivity and inactivation
(DOC)

Text S16 Maximal net modifications on the threshold, the inverse gain and firing frequency
(DOC)

Text S17 Action potential and leak conductance efficacies and modifications
(DOC)
