# Supplementary figures and images for: A Theory of Rate Coding Control by Intrinsic Plasticity Effects
Source: PLoS Comput Biol. 2012 Jan 19;8(1):e1002349. doi: 10.1371/journal.pcbi.1002349 (PMC3261921; doi:10.1371/journal.pcbi.1002349)

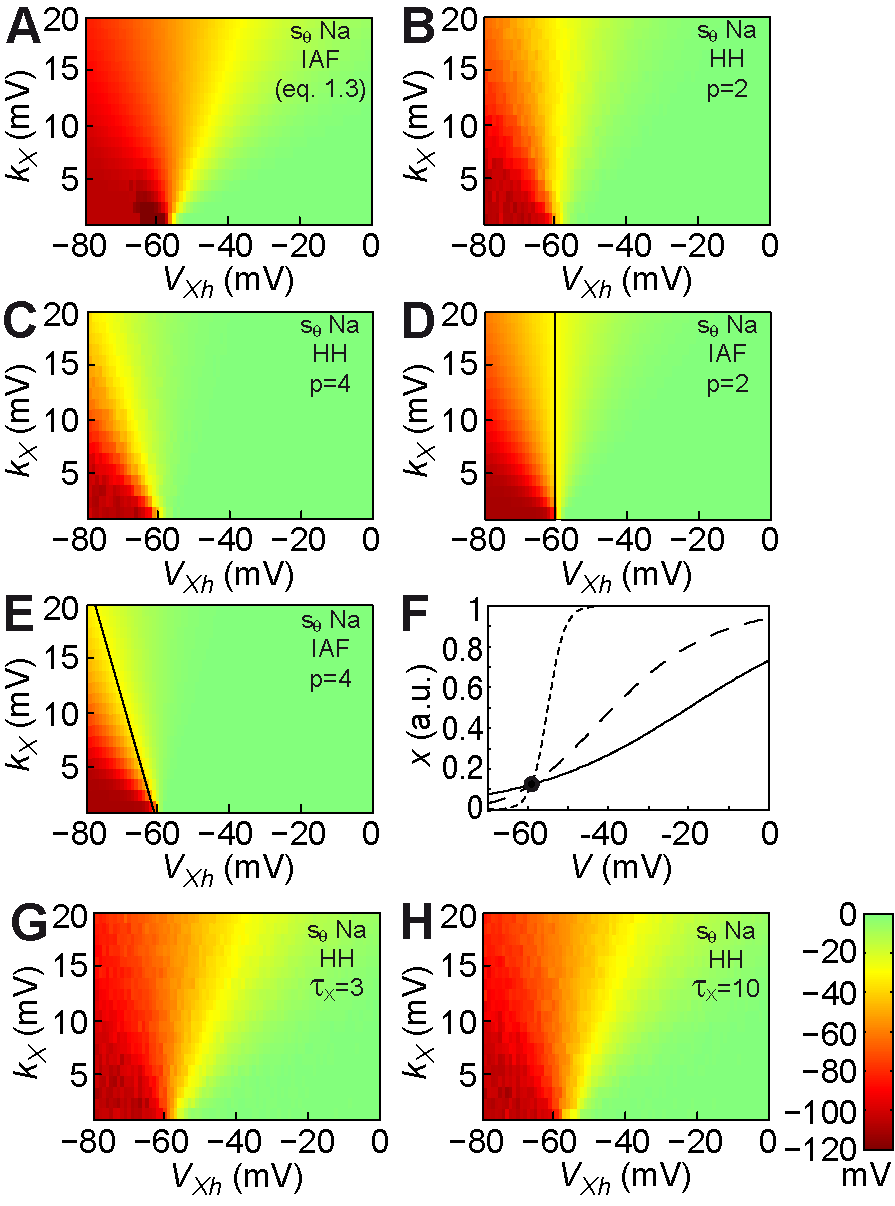

Supplement: Figure S1 — Threshold sensitivity and activation kinetics. (A) Theoretical threshold sensitivity map derived from the threshold IAF theory computed from equation (1.3). (B) Threshold sensitivity map of the standard HH model with. (C) Threshold sensitivity map of the standard HH model with . (D) Theoretical threshold sensitivity map derived from the threshold IAF theory with . Black line: isocline . (E) Theoretical threshold sensitivity map derived from the threshold IAF theory with . Black line: isocline . (F) Activation curves of three conductance with very different , and share the same activation at , and thus the same threshold sensitivity (black dot). Solid line: , , ; dotted line: , , ; dashed line: , , . (G) Threshold sensitivity map of the standard HH model (sodium conductance) with activation time constant . (H) Same as (G), with . (A, B, C, E, G) Colorbar as in (H). (TIF) [file pcbi.1002349.s001.tif]

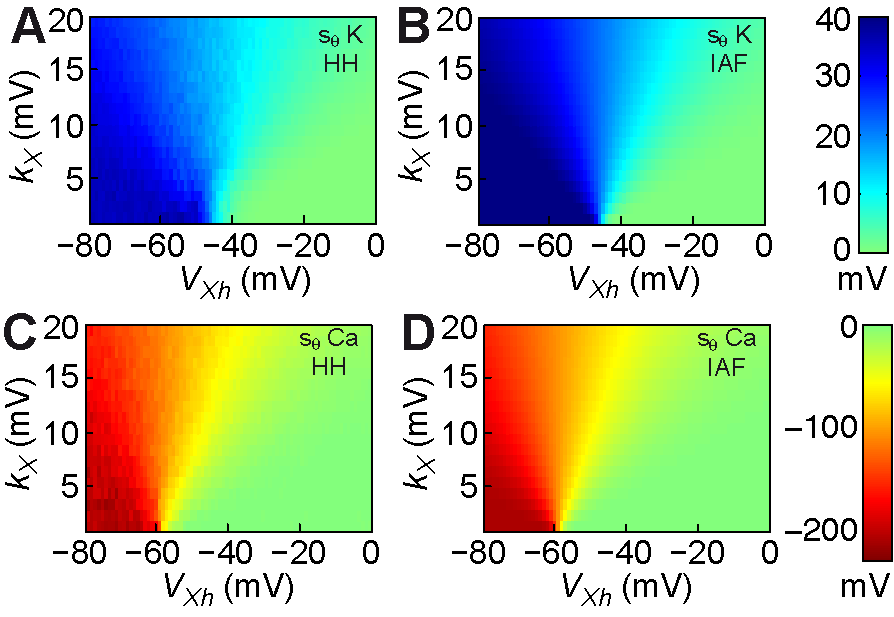

Supplement: Figure S2 — Threshold sensitivity and reversal potential. (A) Threshold sensitivity map of the standard HH model with potassium currents (). Colorbar as in (B). (B) Theoretical threshold sensitivity map derived of the threshold IAF theory with potassium conductance (). (C) Threshold sensitivity map of the standard HH model with calcium conductance (). Colorbar as in (D). (D) Theoretical threshold sensitivity map derived from the threshold IAF theory with calcium conductance (). (TIF) [file pcbi.1002349.s002.tif]

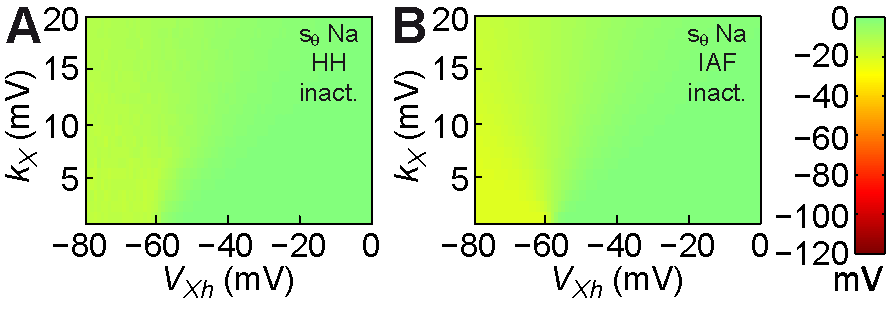

Supplement: Figure S3 — Threshold sensitivity and inactivation. (A) Threshold sensitivity map of the standard HH model in the presence of inactivation (, , ). Colorbar as in (B). (B) Theoretical threshold sensitivity map derived from the threshold IAF theory with inactivation. (TIF) [file pcbi.1002349.s003.tif]

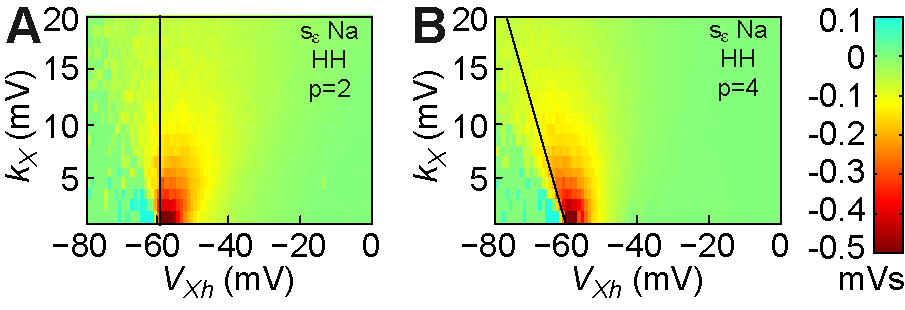

Supplement: Figure S4 — Inverse gain sensitivity and activation power. (A) Inverse efficacy sensitivity map of the standard HH model, . Black line: isocline . Colorbar as in (B). (B) Inverse efficacy sensitivity map of the standard HH model, . Black line: isocline . (TIF) [file pcbi.1002349.s004.tif]

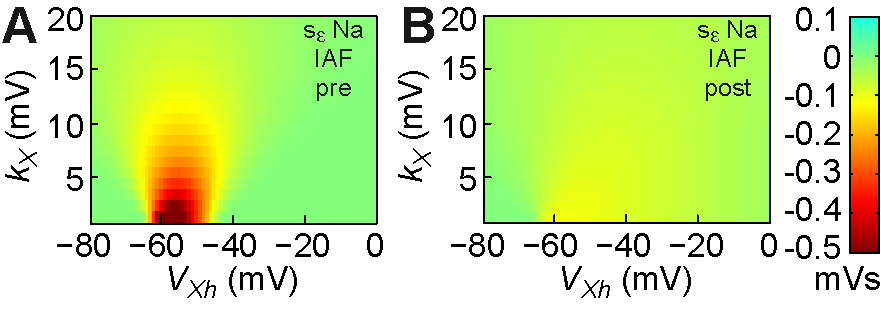

Supplement: Figure S5 — Inverse gain sensitivity in the pre- and post-spike IAF theories. (A) Theoretical inverse gain sensitivity map derived from the post-spike IAF theory with sodium conductance. (B) Theoretical inverse gain sensitivity map derived from the pre-spike IAF theory with sodium conductance. (TIF) [file pcbi.1002349.s005.tif]

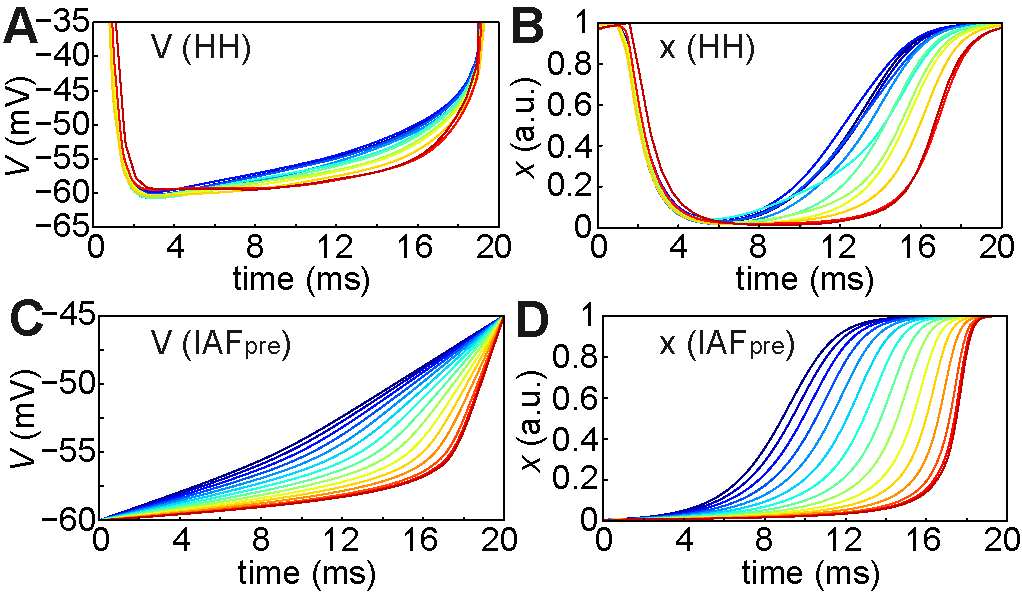

Supplement: Figure S6 — ISI dynamics for a sodium X conductance in the domain of large inverse gain sensitivities. (A) Mean membrane potential dynamics of the standard HH model for increasing with adjusted input currents to match a common firing frequency of 50 Hz. Blue to red curves: with ( and ). (B) X conductance activation dynamics corresponding to (A). (C) Theoretical membrane potential dynamics in the pre-spike IAF theory, with , and similar to (A). (D) Activation dynamics corresponding to (C). (TIF) [file pcbi.1002349.s006.tif]

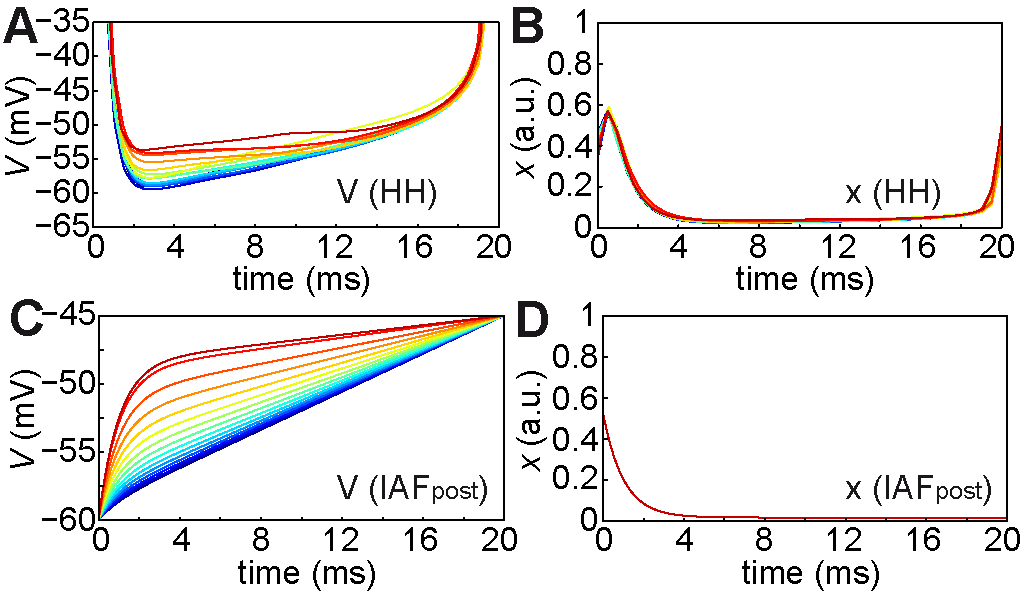

Supplement: Figure S7 — ISI dynamics for a sodium X conductance in the large domain of moderate inverse gain sensitivities. (A) Mean membrane potential dynamics of the standard HH model for increasing with adjusted input currents to match a common firing frequency of 50 Hz. Blue to red curves: with ( and ). (B) X conductance activation dynamics corresponding to (A). (C) Theoretical membrane potential dynamics in the post-spike IAF theory, with , and similar to (A). (D) Activation dynamics corresponding to (C). (TIF) [file pcbi.1002349.s007.tif]

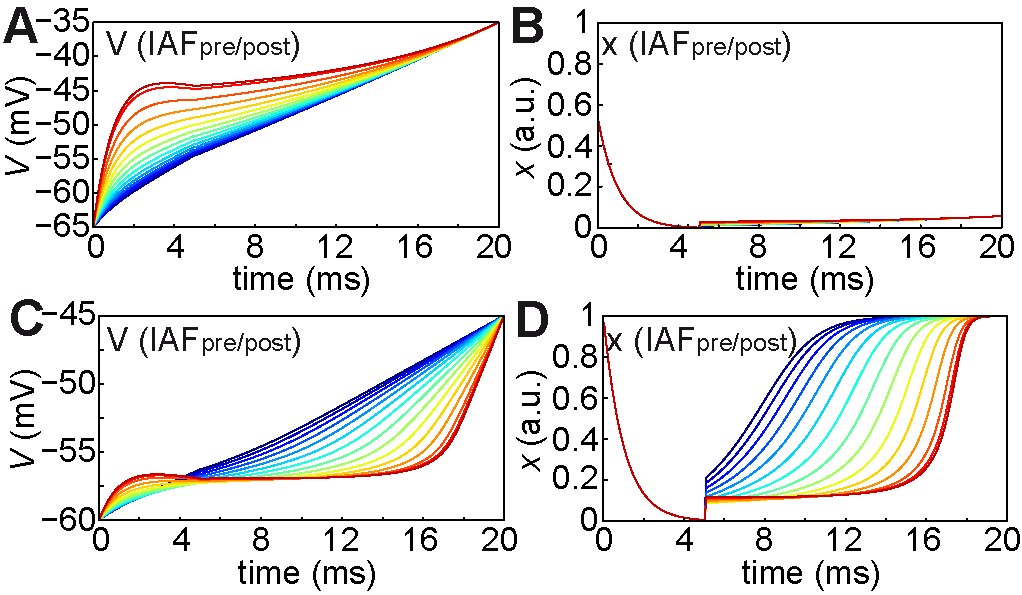

Supplement: Figure S8 — ISI dynamics for a sodium X conductance in the pre/post-spike theory. (A) Theoretical membrane potential dynamics in the pre/post-spike IAF theory, with parameters similar to those used in Figure S6. (B) Activation dynamics corresponding to (A). (C) Theoretical membrane potential dynamics in the pre/post-spike IAF theory, with parameters similar to those used in Figure S7. (D) Activation dynamics corresponding to (C). (TIF) [file pcbi.1002349.s008.tif]

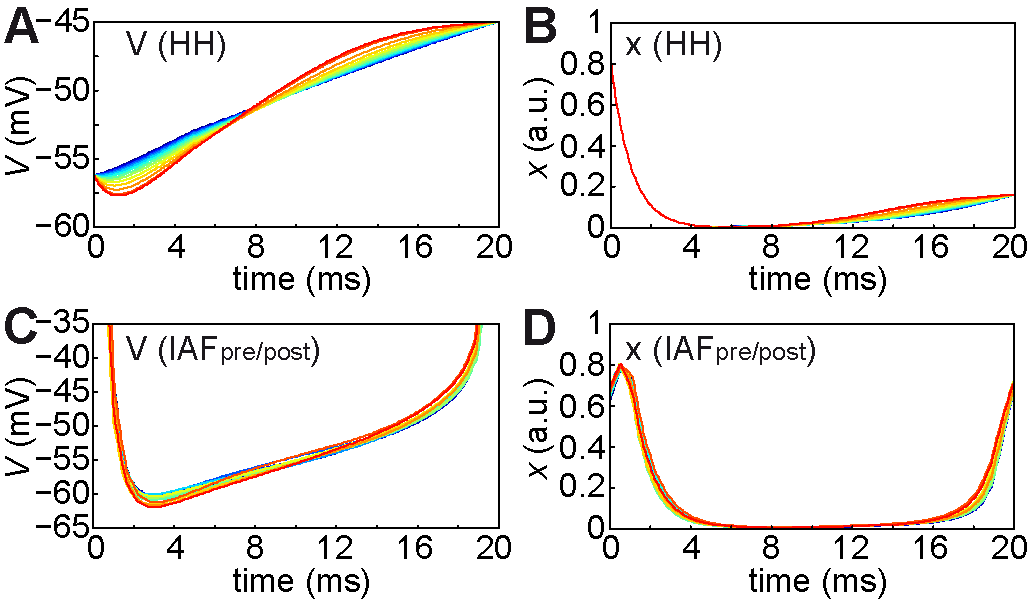

Supplement: Figure S9 — ISI dynamics for a potassium X conductance in the pre/post-spike theory. (A) Mean membrane potential dynamics of the standard HH model with potassium conductance, for increasing with adjusted input currents to match a common firing frequency of 50 Hz. Blue to red curves: with ( and ). (B) X conductance activation dynamics corresponding to (A). (C) Theoretical membrane potential dynamics in the pre/post-spike IAF theory, with , and similar to (A). (D) Activation dynamics corresponding to (C). (TIF) [file pcbi.1002349.s009.tif]

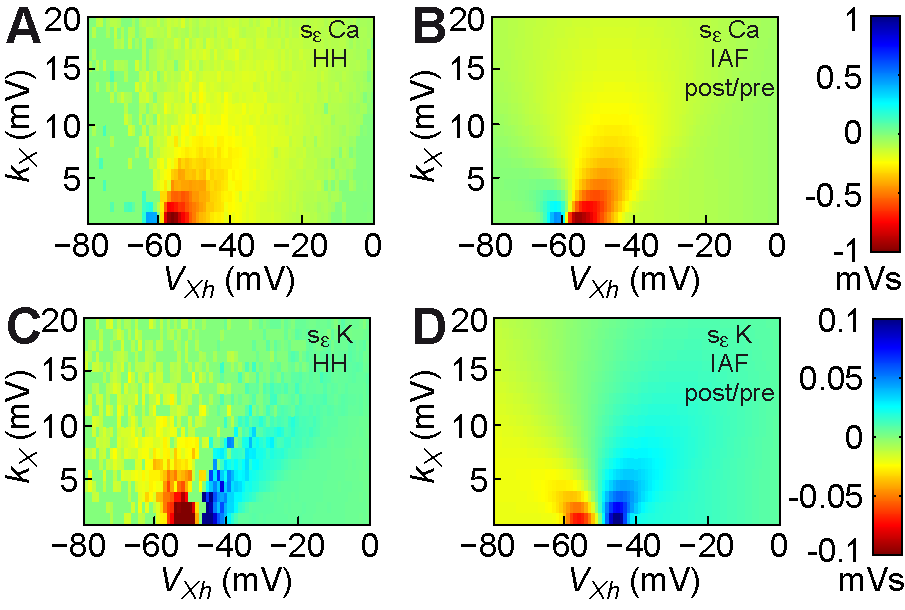

Supplement: Figure S10 — Inverse gain sensitivity and reversal potential. (A) Inverse gain sensitivity map of the standard HH model with calcium conductance. (B) Theoretical inverse gain sensitivity map derived from the pre/post-spike IAF theory with calcium conductance. (C) Inverse gain sensitivity map of the HH model, with potassium conductance. Colorbar as in (D). (D) Theoretical inverse gain sensitivity map derived from the pre/post-spike IAF theory with potassium conductance. , . (TIF) [file pcbi.1002349.s010.tif]

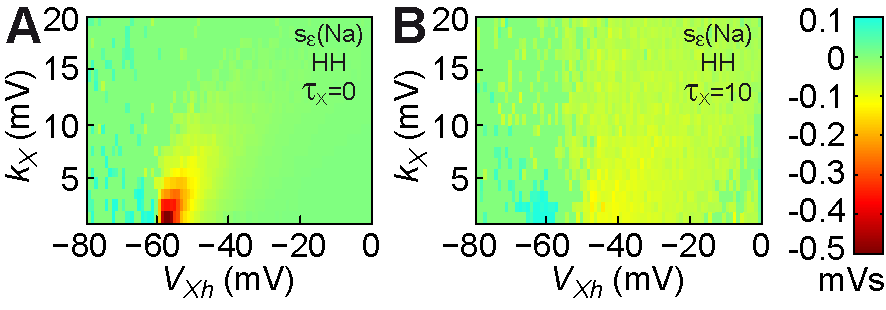

Supplement: Figure S11 — Inverse gain sensitivity and activation time constant. (A) Inverse gain sensitivity map of the standard HH model with instantaneous activation. Colorbar as in (B). (B) Inverse gain sensitivity map of the standard HH model with sodium conductance with activation time constant . (TIF) [file pcbi.1002349.s011.tif]

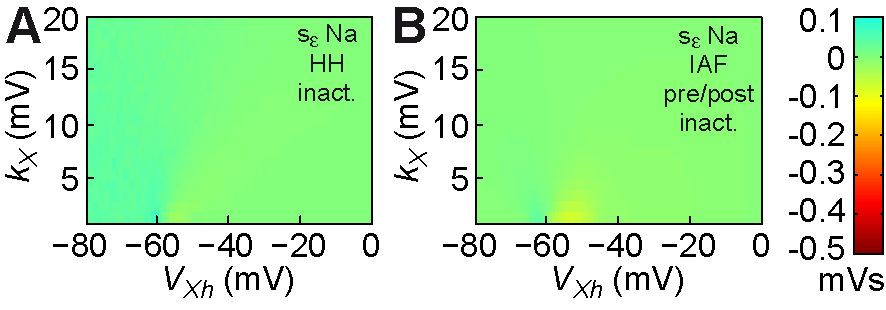

Supplement: Figure S12 — Inverse gain sensitivities and inactivation. (A) Inverse gain sensitivity map of the standard HH model in the presence of inactivation (, , ). Colorbar as in (B). (B) Theoretical inverse gain sensitivity map derived from the pre/post IAF theory with inactivation. (TIF) [file pcbi.1002349.s012.tif]

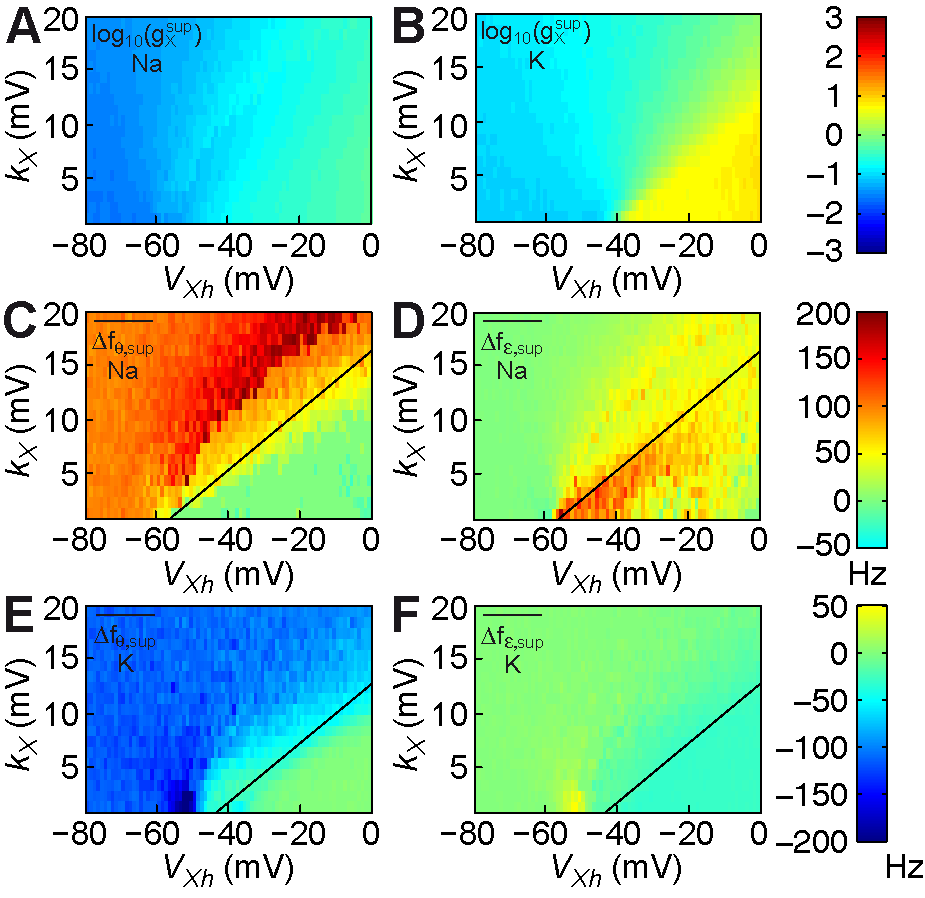

Supplement: Figure S13 — Net frequency effects arising from threshold and inverse gain modulation. (A) Map of the common logarithm of maximal conductance limit for sodium conductance. Colorbar as in (B). (B) Map of the common logarithm of maximal conductance limit for potassium conductance. (C) Net frequency effects from threshold modulation for sodium conductance. Colorbar as in (D). (D) Net frequency effects from inverse gain modulation for sodium conductance. (E) Net frequency effects from threshold modulation for potassium conductance. Colorbar as in (F). (F) Net frequency effects from inverse gain modulation for potassium conductance. (TIF) [file pcbi.1002349.s013.tif]
