# Supplementary material for: A Theory of Rate Coding Control by Intrinsic Plasticity Effects
Source: PLoS Comput Biol. 2012 Jan 19;8(1):e1002349. doi: 10.1371/journal.pcbi.1002349 (PMC3261921; doi:10.1371/journal.pcbi.1002349)
Supplement: Text S1 — The threshold IAF theory (DOC) [file pcbi.1002349.s014.doc]

#### **Text S1. The threshold IAF theory**

In the following, we seek developing a theoretical model to analytically account and interpret the map obtained with HH simulations. We consider an ideal integrate-and-fire (IAF) equivalent version of the standard HH model (see Methods) in which conductance activation is set instantaneous so that an analytical expression of threshold sensitivity can be derived. The membrane potential evolves according to

(1.1)

where $V_{R}$ is the reset potential and $V_{S}$ the onset AP threshold, commonly measured in experiments as the potential corresponding to voltage derivative signaling AP initiation (e.g. $10V/s$; [1]).

The threshold current, or rheobase, $q$, is the injected current above which spiking occurs, i.e. membrane depolarization is insured from $V_{R}$ to $V_{S}$. Injecting $I=q$ upward shifts the (voltage derivative/voltage) relation so that the voltage derivative is strictly positive over except for a single potential at which it is null. This potential, denoted $V_{q}$, represents the effective AP threshold, i.e. the minimal potential above which membrane unconditionally depolarizes, eventually triggering an AP ( reaches ). At $V_{q}$, , so that one derive from equation (1.1)

(1.2),

where $x_{q}^{p}$ stands for steady-state activation at $V_{q}$.

Simulations of the standard HH model showed that the AP threshold was constant () in the absence of the X conductance. By contrast, $V_{q}$ usually varied as a function of $g_{X}$, $V_{\text{Xh}}$ and $k_{X}$, being hyperpolarized in the presence of the sodium conductance. Thus, the threshold sensitivity in the threshold IAF theory expresses as

(1.3).

This expression offered a very good numerical match to the map obtained with HH simulations using averages of $V_{q}$ and over $g_{X}$ (see Methods; Figure S1A). Actually, the correction brought by the second term of the RHS was globally negligible, although it allowed local corrections accounting for the light distortion of the radial structure around the central point and for the small region of overshooting large sensitivity values observed just left from the central point. Hence, a simpler estimate of $s_{q}$ could be considered

(1.4),

which offered an excellent match to the standard HH model, while offering a concise representation of the threshold sensitivity.

Note that the effective AP threshold we study, $q$, does not affect the gain, $e$, contrarily to the phenomenological onset threshold (termed here $V_{S}$) of IAF theories [1]. Thus, the gain, $e$, is not causally dependent upon variations of the threshold, $q$.

**REFERENCES**

1. Platkiewicz J, Brette R (2010) A threshold equation for action potential initiation. PLoS Comput Biol 6: e1000850.
