# Supplementary material for: A Theory of Rate Coding Control by Intrinsic Plasticity Effects
Source: PLoS Comput Biol. 2012 Jan 19;8(1):e1002349. doi: 10.1371/journal.pcbi.1002349 (PMC3261921; doi:10.1371/journal.pcbi.1002349)
Supplement: Text S2 — Threshold sensitivity and activation kinetics (DOC) [file pcbi.1002349.s015.doc]

#### **Text S2. Threshold sensitivity and activation kinetics**

We assessed the generic character of threshold sensitivity properties in the HH model and the IAF theory to modifications of activation kinetic parameters of the X conductance.

Simulations of the HH model with increasing activation power distorted the map leftward (Figure S1B-C). Indeed, higher values yield lower $x_{q}^{p}$ () and hence , producing an apparent leftward shift of the map ( monotonically decreases with higher ). We verified that the theoretical description of $s_{q}$, derived for an arbitrary value of (equation (1.4) in Text S1) accounts for the maps obtained in the HH model for activation power values up to 4 (i.e. a conservative upper bound of in HH models; compare Figure S1D-E with Figure S1B-C). Note that conductance with distinct activation curves (i.e. resulting from different triplets) can exhibit the same , provided that they present the same $x_{q}^{p}$ , i.e. the same activation at (Figure S1F).

Simulations of the HH model with longer activation time constants yielded maps similar to those obtained in the standard HH model (): a radial structure with larger sensitivities occurring for conductance activating sub-threshold (Figure S1G-H). However, the transition between large and null sensitivities at was shallower (Figure S1G-H; consistent, the map with instantaneous activation presented a sharper transition, see Figure 2D). Moreover, the whole map was shifted rightward with larger , so that changes in principally occurred for conductance with steep steady-state activation (low ) and between and (Figure S1G-H). With instantaneous activation (, see Figure 2D), these conductance display a prominent buildup to (see Text S6) during the ISI and each new AP deactivates the conductance back to . Therefore, with larger , incomplete deactivation following the AP increases the mean activation level during the ISI that translate into larger according to equation (1.4) (Text S1). Therefore, our theoretical approach accounts for the apparent rightward shift of the map, as decreases with and is bounded in . Our initial theoretical analysis was thus confirmed in that slower kinetics indeed brings larger in HH simulations (see A formal analysis of firing rate intrinsic plasticity). However, this effect remained limited (<5 mV), even with as large as 10 ms. Thus, it did not change the global conclusion that large threshold sensitivities are typical of conductances with large activation at the threshold potential.

Together, these results demonstrate that the properties of threshold sensitivity are generic across large ranges of activation kinetics of the X conductance.
