# Supplementary material for: A Theory of Rate Coding Control by Intrinsic Plasticity Effects
Source: PLoS Comput Biol. 2012 Jan 19;8(1):e1002349. doi: 10.1371/journal.pcbi.1002349 (PMC3261921; doi:10.1371/journal.pcbi.1002349)
Supplement: Text S3 — Threshold sensitivity and reversal potential (DOC) [file pcbi.1002349.s016.doc]

#### **Text S3. Threshold sensitivity and reversal potential**

Simulations with calcium or potassium reversal potential for the generic X conductance yielded maps with properties similar to that obtained with the standard HH model. For instance, the map obtained with a potassium reversal conductance displayed a radial organization with highest sensitivities for conductance strongly activated at $V_{q}$, i.e. with inferior to $V_{q}$ and small values (Figure S2A). Because potassium conductance are outward, was positive and was depolarized, symmetrical to what was observed for inward sodium conductance. Computing the theoretical map from our analytical expression with led to an excellent match of the HH model $s_{q}$ map for potassium conductance (see Figure S2B). Likewise, using a calcium reversal potential $x_{R}$, the map obtained from HH simulations (see Figure S2C), which was similar to that obtained with a sodium reversal potential, except that it was rescaled by a factor ~2 (compare to Figure S2A), was perfectly matched for by the theoretical map computed from the threshold IAF theory (see Figure S2D). Together, these results indicated that scales the whole map, determining its sign, and that threshold sensitivity is indeed proportional to the driving force of the X current at , as can be predicted from the theoretical expression of (Text S1). Thus, the properties of threshold sensitivity are generic across physiological variations of the reversal potential.
