# Supplementary material for: A Theory of Rate Coding Control by Intrinsic Plasticity Effects
Source: PLoS Comput Biol. 2012 Jan 19;8(1):e1002349. doi: 10.1371/journal.pcbi.1002349 (PMC3261921; doi:10.1371/journal.pcbi.1002349)
Supplement: Text S4 — Threshold sensitivity and inactivation (DOC) [file pcbi.1002349.s017.doc]

#### **Text S4. Threshold sensitivity and inactivation**

We evaluated the impact of inactivation on the maps both in HH simulations and in the theoretical IAF theory. In HH simulations, the threshold sensitivity systematically decreased when the X conductance of the standard model was endowed with an inactivation variable, but left the map structure unaltered. For instance, when the standard HH model (i.e. sodium conductance) was embedded with inactivation (, ; see Methods), we obtained a map that was globally reduced by a factor ~ 10 for (Figure S3A).

In the threshold IAF theory, we considered the hypothesis of constant inactivation during ISI, i.e. , with standing for the steady-state inactivation at the mean membrane potential . We set , assuming linear depolarization from to , a parsimonious approximation when considering the large spectrum of convex/concave membrane potential trajectories that arises across the parameter space. Equation system (1.1) (Text S1) can thus be rewritten

(4.1).

The reasoning employed in the absence of inactivation (see A formal account of threshold sensitivity) holds so that threshold sensitivity can be written as

(4.2).

This theoretical expression perfectly matches the HH map (Figure S3B). Similarly, it provided an excellent match to maps obtained with HH simulations in the case of calcium and potassium conductance or with conductance with different inactivation voltage-dependence ranging the physiological range (not shown). Furthermore, we checked that this decrease in was globally independent of the time constant of inactivation, even with inactivation time constants more than an order of magnitude larger. Actually, even with , variations of the inactivation variable were negligible during the ISI.

Together, these results demonstrated that the properties of threshold sensitivity are robust to the presence of inactivation and well accounted for by the IAF threshold model with a level of inactivation constant during the ISI.
