# Supplementary material for: A Theory of Rate Coding Control by Intrinsic Plasticity Effects
Source: PLoS Comput Biol. 2012 Jan 19;8(1):e1002349. doi: 10.1371/journal.pcbi.1002349 (PMC3261921; doi:10.1371/journal.pcbi.1002349)
Supplement: Text S5 — Inverse gain sensitivity and activation power (DOC) [file pcbi.1002349.s018.doc]

**Text S5. Inverse gain sensitivity and activation power**

We computed maps in the case of the standard model with activation power (Figure S4A) and (Figure S4B). The maps retained the same structure as for , although they were distorted in a way similar to the corresponding maps. Thus, they were separated from domains by the isocline , whatever the activation power considered.
