# Supplementary material for: A Theory of Rate Coding Control by Intrinsic Plasticity Effects
Source: PLoS Comput Biol. 2012 Jan 19;8(1):e1002349. doi: 10.1371/journal.pcbi.1002349 (PMC3261921; doi:10.1371/journal.pcbi.1002349)
Supplement: Text S6 — The pre-spike IAF theory (DOC) [file pcbi.1002349.s019.doc]

#### **Text S6. The pre-spike IAF theory**

Here, we explore the ability of an IAF theory to account for inverse gain modifications, based on the build up of activation before each forthcoming AP. To incarnate this mechanism, we assume that activation of the X conductance has instantaneous dynamics, i.e. . Depending on and , this build up may be almost nearly flat or strong and rapid. We also assume that and that the driving forces and are taken constant. Under these assumptions, the autonomous ODE

(6.1)

can be analytically integrated, yielding

(6.2),

where is the ISI duration, , , and denote the threshold current corresponding to activation levels 0, 1, and , respectively. Thus,

(6.3).

Because the firing frequency is a ratio of two functions of the input current , one can compute . After some algebra, one shows that the inverse gain can be written as

(6.4)

Note that for $g_{X}=0$, the term , so that . Computing is truly cumbersome, so we could not reach its exact analytical formulation. However, because $e$ is nearly linear with $g_{X}$ (see Threshold and inverse gain linearly depend on $g_{X}$ in the Results section), i.e. , we computed , i.e.

(6.5)

to estimate the inverse gain sensitivity. We computed at , where was the current threshold of the pre-spike IAF theory and a constant input current. In the pre-spike IAF theory, the voltage threshold (as defined in the threshold IAF theory, Text S1) is in the case of excitatory conductance. Indeed, the voltage-dependence of the voltage-derivative is entirely determined by (see equation 6.1), which implies that the largest input current that has to be applied to shift the entire relation to positive values corresponds to the potential at which activation is minimal, i.e. . In the case of an inhibitory X conductance, so that one gets for symmetrical reasons. The estimation of we obtained was robust with regard to the exact value of . In the figures, the input current used in the pre-spike IAF theory is the same as the one derived from the pre/post-spike IAF theory (typically ).

As can be noted, this expression indeed depends on the difference between and and logarithms thereof. Computing the $s_{e}$ map demonstrated that the build-up effect characterizing the pre-spike IAF theory accounted for the peak of large sensitivities surrounding O (Figure S5A; compare with Figure 3A). Conductance in this region present ranging between $V_{R}$ and $V_{S}$, with small , so that and . Therefore, activation buildup is maximal before each AP, so that the theoretical $s_{e}$ is large from equation (6.5), accounting for the large values of inverse gain sensitivity observed in HH simulations. By contrast, for conductance with above $V_{S}$ or below $V_{R}$, the difference between and almost zeroes, so that is virtually null.

We checked for conductance situated in the peak of large sensitivities surrounding O, that activation in HH simulations indeed displayed a prominent buildup, as well as the similar convex membrane potential trajectory (Text S7). However, the pre-spike IAF theory was unable to account for the large domain of moderate inverse gain sensitivities, indicating that it was not sufficient per se to account for the whole $s_{e}$ map observed in HH simulations (Figure 3A).
