# Supplementary material for: A Theory of Rate Coding Control by Intrinsic Plasticity Effects
Source: PLoS Comput Biol. 2012 Jan 19;8(1):e1002349. doi: 10.1371/journal.pcbi.1002349 (PMC3261921; doi:10.1371/journal.pcbi.1002349)
Supplement: Text S7 — Comparison of dynamics in the standard HH and pre-spike IAF theory (DOC) [file pcbi.1002349.s020.doc]

Text S7. Comparison of dynamics in the standard HH and pre-spike IAF theory.

We checked, in the domain of large inverse gain sensitivities obtained for sodium conductance, that dynamics in the pre-spike IAF theory are consistent with those of the standard HH theory. To do so, we have compared the membrane potential and activation dynamics of a X conductance for increasing values of , adjusting the input current I to compare traces with identical ISI durations. We found that in HH simulations, as for traces obtained with a conductance situated on the large domain of moderate inverse gain sensitivities, activation first deactivates (Figure S6B), depolarizing the potential during the initial part of the ISI with increasing (Figure S6A). However, a prominent activation buildup occurs afterwards (Figure S6B). With increasing , the injected current is smaller, and the influence of the X conductance is larger. The buildup is stronger and arises at later times from the non-linear positive feedback between membrane potential and activation (Figure S6B). As a consequence, the voltage trajectory is more convex (Figure S6A). In the pre-spike IAF theory, the initial deactivation is absent (by construction) and the detailed quantitative match is impossible due to the strong simplifying hypotheses assumed. In particular, activation is instantaneous whereas it has a time-constant of 1 ms in the standard HH model, which explains why activation buildup precedes that observed in HH simulations. However, the prominent buildup, its delay with increasing , as well as its consequences on the convex form of the membrane potential trajectory were correctly captured by the post-spike IAF model (Figure S6C-D).
