# Supplementary material for: A Theory of Rate Coding Control by Intrinsic Plasticity Effects
Source: PLoS Comput Biol. 2012 Jan 19;8(1):e1002349. doi: 10.1371/journal.pcbi.1002349 (PMC3261921; doi:10.1371/journal.pcbi.1002349)
Supplement: Text S8 — The post-spike IAF theory (DOC) [file pcbi.1002349.s021.doc]

#### **Text S8. The post-spike IAF theory**

This theory was built to express the ideal scenario where the activation of the X conductance is entirely determined by its relaxation following the AP. We assume that and formulate the hypothesis that the activation time constant is significantly faster than the membrane time constant (). Thus, decreases exponentially toward its steady-state value before significantly differs from its initial condition , so that dynamics can be approximated as

(8.1)

where is the value attained at the end of the AP (i.e. the initial condition for ; see Text S9) and with $V_{R}$ the resting potential. Thus, the IAF post-spike theory can be written as

(8.2)

To analyze this model analytically, we had to fix the driving forces of the currents to constant values. In the following, we denote and , where . Under these assumptions, the non-autonomous ODE

(8.3)

can be analytically integrated so that

(8.4)

For time constants of a few milliseconds, one has (the ISI duration ranges in our HH simulations), so that . Under these conditions,

(8.5),

so that

(8.6)

and

(8.7).

Finally, one obtains

(8.8).

Computing this expression in the plane provides a sensitivity map that accounts for the large domain of moderate inverse gain sensitivities (Figure S5B; compare with Figure 3A). In that region, is small (activation is steep) and lies between and the AP half-height potential. As a consequence, nearly zeroes and is large, so that the theoretical $s_{e}$ is large from equation (8.8). Moreover, we checked in this domain that membrane potential and activation dynamics were consistent with those of the standard HH model (Text S10). However, the post-spike IAF theory did not account for the prominent peak of large inverse gain sensitivities (Figure 3A). Thus, a theory where activation dynamics is solely described by deactivation following AP occurrence is not sufficient to account for inverse gain sensitivities across the map in the standard HH model.
