# Supplementary material for: A Theory of Rate Coding Control by Intrinsic Plasticity Effects
Source: PLoS Comput Biol. 2012 Jan 19;8(1):e1002349. doi: 10.1371/journal.pcbi.1002349 (PMC3261921; doi:10.1371/journal.pcbi.1002349)
Supplement: Text S9 — Activation dynamics during the spike (DOC) [file pcbi.1002349.s022.doc]

**Text S9. Activation dynamics during the spike**

The post-spike and pre/post-spike IAF models require to determine activation of the X conductance upon AP termination, , as it represents the initial condition for the following ISI. To obtain a tractable analytical estimation of , we formulate the following hypotheses. First, activation has reached its steady-state value when the membrane potential crosses the onset AP threshold . Second, the membrane potential dynamics can be sketched as a linear depolarization from to , the peak membrane potential, followed by a linear repolarization from to , the reset membrane potential. Third, activation converges with first-order kinetics toward , the mean steady-state activation during the AP.

In the following, we denote the AP duration, i.e. the time required to depolarize from to and to repolarize from to . We also denote the duration of depolarization and that of repolarization. Thus , and . As seen later, the exact value of has no importance on calculations.

The mean steady-state activation during the AP is

(9.1)

Writing membrane potential dynamics in two distinct time-bases for the depolarization and the repolarization phases with time 0 corresponding membrane potential and , one can write:

. (9.2)

Such functions can be integrated, as following,

,

so that after some algebra, one obtains

, (9.3)

which does not depend on , or . From this result and hypotheses 1) and 3), one directly computes , the activation reached at the end of the AP, which can be written as

, (9.4)

where represents the average time-constant of the X conductance at potentials superior to . We distinguish from in our theory, to catch in a simple way the possible voltage-dependence of activation time-constants that is found for some conductance empirically studied in vitro. We took to compute maps, but their values could be set to different values when computing .
