# Supplementary material for: A Theory of Rate Coding Control by Intrinsic Plasticity Effects
Source: PLoS Comput Biol. 2012 Jan 19;8(1):e1002349. doi: 10.1371/journal.pcbi.1002349 (PMC3261921; doi:10.1371/journal.pcbi.1002349)
Supplement: Text S10 — Comparison of dynamics in the standard HH and post-spike IAF theory (DOC) [file pcbi.1002349.s023.doc]

Text S10. Comparison of dynamics in the standard HH and post-spike IAF theory.

We have checked, in the large domain of moderate inverse gain sensitivities obtained for sodium conductance, that dynamics in the post-spike IAF theory were consistent with those of the standard HH model. To do so, we have compared the membrane potential and activation dynamics of the X conductance for increasing values of , adjusting the input current I to compare traces with identical ISI durations. We found in HH simulations that activation essentially deactivated toward a relatively constant value during the ISI (Figure S7B; the activation buildup at the end of the trace does not occur during the ISI but during the next spike). This initial deactivation depolarized the membrane potential in the first part of the ISI more and more with increasing (Figure S7A). Obviously, because of the strong simplifying hypotheses we have done in the post-spike IAF theory (neglecting the spike mechanism, imposing fixed reset and threshold potentials), the match with HH simulations is not expected to be quantitatively exact. However, essential dynamical characteristics of HH simulations were found in the post-spike IAF theory. Indeed, initial deactivation is a constitutive feature of the post-spike IAF theory (Figure S7D). Moreover, the membrane potential rapidly converged to increasingly depolarized potentials during the first part of the ISI with increasing , due to the depolarizing influence of the X conductance, and the membrane potential then converged toward $V_{S}$from these different potentials, as in as in HH simulations (Figure S7C).
