# Supplementary material for: A Theory of Rate Coding Control by Intrinsic Plasticity Effects
Source: PLoS Comput Biol. 2012 Jan 19;8(1):e1002349. doi: 10.1371/journal.pcbi.1002349 (PMC3261921; doi:10.1371/journal.pcbi.1002349)
Supplement: Text S11 — The pre/post-spike IAF theory (DOC) [file pcbi.1002349.s024.doc]

#### **Text S11. The pre/post-spike IAF theory**

To get a global account of the inverse gain sensitivity, we devised the pre/post-spike IAF theory, which combines activation dynamics of the post-spike IAF theory with those of the pre-spike IAF theory. Specifically, in the pre/post-spike IAF theory, the ISI is divided in two consecutive periods. The membrane potential dynamics is governed first by the pre-spike IAF theory (see equation 6.1, Text S6) in the time interval , then by the pre-spike IAF theory (see equation 8.4, Text S8) in the time interval , where is the ISI duration. We denote the membrane potential at that time . We set , i.e. determines the relative number of activation time constants during which activation exponentially relaxes toward (larger values result in a larger relative influence of the post-spike IAF theory during the ISI). From equation (8.4), one can determine :

(11.1),

The membrane potential is taken as the initial condition of the pre-spike IAF theory, to determine the time required reaching , which is obtained from equation (6.2) by replacing by :

(11.2),

where denotes the steady-state activation at , , and are those of the post-spike IAF theory and . Thus, the firing frequency expresses as

(11.3).

The firing frequency has the form , so one can compute . After some algebra, one shows that the inverse gain can be written as

(11.4).

For $g_{X}=0$, the term , so that . Again, computing is cumbersome, so we computed , i.e.

(11.5)

to estimate its value. The input current was determined as in the pre-spike IAF theory. The mean voltage potential was , being a fraction so that . Best fits of the landscape were obtained with input currents in the range , and for values in the range .
