# Supplementary material for: A Theory of Rate Coding Control by Intrinsic Plasticity Effects
Source: PLoS Comput Biol. 2012 Jan 19;8(1):e1002349. doi: 10.1371/journal.pcbi.1002349 (PMC3261921; doi:10.1371/journal.pcbi.1002349)
Supplement: Text S12 — Comparison of dynamics in the standard HH and pre/post-spike IAF theory (DOC) [file pcbi.1002349.s025.doc]

Text S12. Comparison of dynamics in the standard HH and pre/post-spike IAF theory.

As for the pre-spike and post-spike theories, an exact quantitative match with the standard HH model cannot be expected, due to model simplifications, especially considering the combination of both models at a fixed time in the pre/post IAF model (note also that activation buildup anticipated that observed in HH simulations, due to instantaneous activation, as in the pre-spike model). However, we found that the traces obtained from the pre/post IAF model captured the essential dynamical features of HH simulations, both in the large domain of moderate inverse gain sensitivities and in the domain of large sensitivities.

Indeed, in the domain of large inverse gain sensitivities, the trajectories of the voltage and activation of the X conductance (Figure S8C-D) similarly conserved the properties of the pre-spike model (Figure S6C-D) and thus still accounted for those of the standard HH model (Figure S6A-B). Again, the dynamics were better captured with the pre/post model, as it accounted both for the initial deactivation phase of the conductance (compare Figure S8D and Figure S6B), as well as for the consequent depolarization that leads to the crossing of voltage trajectories (compare Figure S8C and Figure S6A).

In the large domain of inverse gain sensitivity, the trajectories of the voltage and activation of the X conductance (Figure S8A-B) resembled those of the post-spike IAF theory (Figure S7C-D) and accounted well for HH simulations (Figure S7A-B). Moreover, the pre/post-spike model even better captured the voltage dynamics, as it accounted for the initial concave character of the voltage trajectory (compare Figure S8A and Figure S7A) and for the slight activation buildup found during the second part of the ISI (compare Figure S8B and Figure S7B).

In the case of potassium conductance, we also found that the pre/post-spike IAF theory was able to reproduce the cardinal dynamical properties observed in HH simulations. Indeed, in the domain of positive inverse gain sensitivity, the voltage trajectories present a crossing that opposes that observed for sodium conductance: with increasing the initial potential hyperpolarize, while this is the contrary just before spiking (Figure S9A). The pre/post-spike IAF theory reproduced well that feature (Figure S9C), as well as the deactivation and build up of the X conductance activation (compare Figure S9D and Figure S9B).
