# Supplementary material for: A Theory of Rate Coding Control by Intrinsic Plasticity Effects
Source: PLoS Comput Biol. 2012 Jan 19;8(1):e1002349. doi: 10.1371/journal.pcbi.1002349 (PMC3261921; doi:10.1371/journal.pcbi.1002349)
Supplement: Text S13 — Inverse gain sensitivity and reversal potential (DOC) [file pcbi.1002349.s026.doc]

#### **Text S13. Inverse gain sensitivity and reversal potential**

We checked that the pre/post-spike IAF theory accounted for the inverse gain sensitivity maps in the case of conductance with different reversal potentials.

In the case of calcium conductance, the inverse gain sensitivity map obtained with the HH model displayed a structure similar to that obtained for sodium currents, except for a twofold increase of absolute values of $s_{e}$ (Figure S10A). Using the theoretical expression derived from the pre/post-spike IAF theory with the calcium reversal potential yielded a theoretical inverse gain sensitivity map that fully accounted for that obtained with HH models (Figure S10B). The dynamics of membrane potential and activation of the X conductance were similar to those obtained from simulations with sodium conductance (not shown).

In the case of potassium conductance, the inverse gain sensitivity map presented a global structure that was inverted compared to that of sodium conductance, consistent with the opposite sign of the driven force of the X conductance (Figure S10C). However, the relative size of the paradoxical peak of negative inverse gain sensitivity (i.e. increased gain, increased excitability) was larger than that observed for inward conductance and its amplitude was equal to the peak of “normal” inverse gain sensitivities. In that domain, the negative feedback between membrane potential and the conductance activation imposed very slow depolarization in the last millivolts beneath the AP threshold. This dampening effect thus lasted much longer at small firing frequencies, increasing the gain. Despite the inverse gain sensitivity map strongly departs from that obtained with sodium conductance, the pre/post-spike IAF theory offered an excellent account of it (Figure S10D), suggesting that it captures essential features of the causal mechanisms determining inverse gain sensitivity. Indeed, we verified that dynamics of the membrane potential and activation were correctly accounted for by the pre/post-spike IAF theory (see Text S12).
