# Supplementary material for: A Theory of Rate Coding Control by Intrinsic Plasticity Effects
Source: PLoS Comput Biol. 2012 Jan 19;8(1):e1002349. doi: 10.1371/journal.pcbi.1002349 (PMC3261921; doi:10.1371/journal.pcbi.1002349)
Supplement: Text S14 — Inverse gain sensitivity and activation time-constant (DOC) [file pcbi.1002349.s027.doc]

#### **Text S14. Inverse gain sensitivity and activation time-constant**

Simulations of the standard HH model with different activation dynamics confirmed the conclusions drawn from the pre/post-spike IAF theory. Indeed, with instantaneous activation, the large domain of moderate $s_{e}$ was absent whereas the domain of large sensitivity remained present (Figure S11A). This result was consistent with our understanding of the mechanisms underlying inverse gain sensitivity. Hence, with instantaneous activation (), deactivation is immediate so that the pre/post-spike IAF theory behaves as the pre-spike IAF theory, which is responsible for the large inverse gain sensitivities but does not account for the large domain of moderate sensitivities.

Computing the inverse gain sensitivity with time constants larger than that of the standard HH model led to opposed situation: the large domain of moderate $s_{e}$ remained present whereas the domain of large sensitivity was absent (Figure S11B). The pre/post-spike IAF theory could not reproduce this effect since its analytical integration requires a version of the post-spike theory with small time-constants (i.e. milliseconds). Nevertheless, the disappearance of the domain of large sensitivity could be anticipated from the fact that larger time-constants slow the post-spike deactivation and thus reduce the pre-spike buildup. Similarly, the stability of the large domain of moderate sensitivities at intermediate could be interpreting as resulting from two opposing effects. Indeed, on the one hand, in the post-spike theory, increases linearly with at small (i.e. ~1ms; see equation (8.8), Text S8). However, on the other hand, converges to 0 in the limit of large . Indeed, at large values, , thus on get

(14.1)

from equation (8.6) (Text S8), so that

(14.2)

and

(14.3).
