# Supplementary material for: A Theory of Rate Coding Control by Intrinsic Plasticity Effects
Source: PLoS Comput Biol. 2012 Jan 19;8(1):e1002349. doi: 10.1371/journal.pcbi.1002349 (PMC3261921; doi:10.1371/journal.pcbi.1002349)
Supplement: Text S15 — Inverse gain sensitivity and inactivation (DOC) [file pcbi.1002349.s028.doc]

#### **Text S15. Inverse gain sensitivity and inactivation**

The pre/post-spike IAF theory could be extended to inactivation by considering constant inactivation during ISI, i.e. . As for the threshold IAF theory, integrating differential equations of the post-spike, pre-spike and pre/post-spike IAF theories remains unchanged because operates as a multiplicative coefficient of the X current driving force. Hence, the inverse gain sensitivity becomes

(14.1)

in the post-spike IAF theory and equations (6.5) and (11.5) remain the same for the pre- and pre/post-spike IAF theories, with the sole difference that threshold currents incorporate the inactivation variable: , , and . The inactivation parameters where similar to those described in the threshold sensitivity inactivation section.

When the X conductance was endowed with an inactivation variable (see Methods), we found in simulations of the standard HH model that the inverse gain sensitivity was systematically decreased, leaving the global map structure unaltered, similar to the change observed for the threshold sensitivity (Figure S9A). We found that the theoretical expression (14.1) provided an excellent account of the map obtained with HH simulations (Figure S9B). Moreover, this theory provided an excellent match to $s_{e}$ maps obtained with HH simulations in the case of calcium and potassium conductance or with conductance with different inactivation voltage-dependence ranging the physiological range (not shown). Furthermore, as for
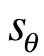
, we checked that this decrease in was globally independent of the time constant of inactivation, even with inactivation time constants more than an order of magnitude larger.
