# Supplementary material for: A Theory of Rate Coding Control by Intrinsic Plasticity Effects
Source: PLoS Comput Biol. 2012 Jan 19;8(1):e1002349. doi: 10.1371/journal.pcbi.1002349 (PMC3261921; doi:10.1371/journal.pcbi.1002349)
Supplement: Text S16 — Maximal net modifications on the threshold, the inverse gain and firing frequency (DOC) [file pcbi.1002349.s029.doc]

#### **Text S16. Maximal net modifications on the threshold, the inverse gain and firing frequency**

Effective modifications of firing frequency by IP (; in response to a given input) originate from net changes of the threshold () or inverse gain () of the **,** or both. Such net variations express as the simple product of sensitivities by conductance variations ()**,** because of the linearity we have observed in HH simulations. They are interesting because one may exist domains of large sensitivity where low  values produce limited net threshold or inverse gain modifications. By contrast, large  in domains of low sensitivity might produce large net modifications. In both case, this would lower the significance of domains of sensitivity. Unfortunately, the regulation of  by IP rules is both extremely diverse (e.g. homeostatic or not) and remains quantitatively unknown in detail hitherto to our knowledge. Thus, estimating actual  resulting from IP rules’ induction by physiological neural activity in vivo currently remains purely speculative.

To the specific exception of the section assessing the possible role of the IP loop on spontaneous firing, the present study does not rely on any particular hypothesis on how IP rules may modify , as we focus on IP effects. However, the progression of values used to determine sensitivities is bounded, because we have set limits for the and the AP duration to keep these electrophysiological parameters within in vivo physiological ranges (see Methods). For any given X conductance (i.e. specified by its biophysical parameters) one of these limits is attained first and we denote this value . Thus, maps can be built. Such maps are important because they set maximal orders of magnitude defining the physiological situation in our study. We found that in the standard HH model,  values are rather low for sodium (and calcium, not shown) conductance (; Figure S13A), compared to potassium conductance, which for  can reach values up to  (Figure 13B). This arises because outward currents, in contrast with inward currents, oppose to the injected current, so that un-physiological excitability requires larger maximal conductance to be reached. Moreover, in both case, we found that  was higher for conductance with large and small , i.e. strictly supra-threshold, compared to sub-threshold conductance, because they activate only correlative to AP occurrence and not during the whole ISI.

Maximal net modifications of threshold and inverse gain can be obtained by multiplying the corresponding sensitivities with $g_{X}^{\text{sup}}$, the limit superior value that can be used without attaining one of the electrophysiological limits (see Methods):

(16.1)

and

(16.2).

To get an overall estimate of the influence of the net modification of the threshold and inverse gain, we proceeded as follows. The modification of a given frequency can be written as

(16.3),

which splits into two groups of frequency modification that relate to the net threshold and inverse gain modifications:

(16.4).

Note that these two groups may have the similar or opposite signs, depending on that of sensitivities. Estimating the mean variation in frequency requires integrating these variations over the whole frequency range considered (i.e.). Thus, one easily obtains

(16.5)

where . Note than again, the mean variation in frequency due to IP can be spitted in two terms that specifically depend on the modifications of threshold and gain:

(16.6),

where and . Note that the same reasoning (equations (16.1) to (16.6)) is similar for any conductance , i.e. one have and .

For sodium conductance, we found that and maps present domains of large frequency modification that globally respect the corresponding and map structures: the domain of large situates at more sub-threshold than that of large (Figure S13C and Figure S13D). However, both domains present a significant shift toward supra-threshold conductance, because they display larger values. Thus, conductance with very low can possibly give rise to large frequency modifications without unphysiological consequence, because is large (e.g. , , Figure S13A and Figure S13C). We found that the two domains of large frequency modifications were grossly separated by the isocline (black line, Figure S13C and Figure S13D), i.e. an extremely low level of activation at the effective threshold potential . For potassium conductance, the shift of the domains of large and large , as well as the localization of their frontier, were globally similar (Figure S13E and Figure S13F). Globally, net frequency effects due to threshold modifications were significantly larger than those induced by inverse gain changes, with a ratio ~1:3 (in the frequency range 0-100 Hz).
