# Supplementary material for: A Theory of Rate Coding Control by Intrinsic Plasticity Effects
Source: PLoS Comput Biol. 2012 Jan 19;8(1):e1002349. doi: 10.1371/journal.pcbi.1002349 (PMC3261921; doi:10.1371/journal.pcbi.1002349)
Supplement: Text S17 — Action potential and leak conductance efficacies and modifications (DOC) [file pcbi.1002349.s030.doc]

**Text S17. Action potential and leak conductance efficacies and modifications**

In some empirical studies, IP has been shown to imply modifications of the sodium or potassium conductance of the AP or of the leak conductance. We have thus assessed the threshold and inverse gain sensibilities and the net associated frequency modifications of these conductance. To do so, we have used the same protocol as for other conductance, except they were added to the basic HH model conductance (**,,**).

For the fast-inactivating sodium conductance, we found , a rather small value. However, the associated mean maximal frequency modification was significant, 20 Hz, because was huge. Note that in this case, we increased the maximal conductance of the HH model sodium conductance, but there was no additional X conductance. Therefore, in the threshold IAF theory (Text S1), the threshold sensitivity becomes in equation (1.3) (Text S1), because the derivative is not minute anymore in that case, accounting for the negative value obtained ( decreased with increasing ). The associated inverse gain frequency was even smaller , leading to a small mean maximal frequency modification ~ -3Hz. The sodium conductance activation is extremely fast (instantaneous in the present model), so that it activates just before the AP and deactivates immediately afterward. Thus, the deactivation and buildup mechanisms responsible for inverse gain changes were virtually null, accounting for the extremely small .

For the potassium conductance, we found a null threshold sensitivity. This result can easily be interpreted in the perspective of threshold IAF theory, as for the sodium conductance (equation (1.3), Text S1). Indeed, because this conductance is very supra-threshold, its effect on is virtually null (), i.e. . The inverse gain sensitivity was , yielding a mean maximal frequency modification ~ -11Hz. The deactivation mechanism accounted entirely for this result, because the potassium conductance deactivates with a time constant > 1 ms and there is no buildup, the conductance being very supra-threshold.

In the case of the leak conductance, we found , which yielded a strong mean maximal frequency modification of ~ -52Hz. This could be expected, as this conductance acts permanently at sub-threshold potentials. The leak conductance can be view as a conductance with a voltage-independent activation . Moreover, we found in that case that the voltage threshold, , is independent of . Thus, one finds using equation (1.4) (Text S1). Finally, was minute (mean maximal frequency modification ~-1Hz), and was accounted for by the fact that deactivation or buildup mechanisms are absent in that case. Note that the threshold modifications we observe here are independent of distinct modifications that have been unravelled following the manipulation of other factors influencing the membrane input resistance, such as, e.g. gain changes in synaptic conductance under conditions of balanced synaptic inputs [1,2].

Thus, sensitivities for these three conductance were well accounted for by the general framework we have developed.

**REFERENCES**

1. Silver RA (2010) Neuronal arithmetic. Nat Rev Neurosci 11: 474-489.

2. Destexhe A, Rudolph M, Pare D (2003) The high-conductance state of neocortical neurons in vivo. Nat Rev Neurosci 4: 739-751.
